# Supplementary material for: Rigidly flat-foldable class of lockable origami-inspired metamaterials with topological stiff states
Source: Nat Commun. 2022 Apr 5;13:1816. doi: 10.1038/s41467-022-29484-1 (PMC8983707; doi:10.1038/s41467-022-29484-1)
Supplement: Supplementary file 1 — Supplementary Information [file 41467_2022_29484_MOESM1_ESM.pdf]

Supplementary Information for

Rigidly flat-foldable class of lockable origami-inspired metamaterials with topological stiff states

Amin Jamalimehr[1,♣], Morad Mirzajanzadeh[1♣], Abdolhamid S. Akbarzadeh[1,2], Damiano Pasini[1,*]

[1] Department of Mechanical Engineering, McGill University, Montreal
[2] Department of Bioresource Engineering, McGill University, Montreal

♣These authors contributed equally

* Corresponding author: Damiano Pasini (e-mail: damiano.pasini@mcgill.ca)

E-mail address for authors: amin.jamalimehr@mail.mcgill.ca, morad.mirzajanzadeh@mail.mcgill.ca, hamid.akbarzadeh@mcgill.ca, damiano.pasini@mcgill.ca

This PDF file includes:

Supplementary Discussions S1 to S11
Supplementary Figures 1 to 14
Supplementary Tables 1 to 3
Supplementary References

Supplementary Discussion

S1. Kinematic analysis

To study the mobility of our unit kinematic chain (Supplementary Fig. 1**a**), we use a surrogate bar network where the planar faces are replaced with pin-jointed bars (Supplementary Fig. 1**b**, **c**). This assembly consists of n_b inextensible bars, n_j pin joints and rigid triangular panels; quad panels are replaced by a set of two connected rigid triangles, thus eliminating in-plane shear deformation. The rigidity of each quad, or triangular panel, is ensured by imposing two constraints: (i) bar inextensibility and (ii) panel coplanarity for the triangular faces making up a quad panel (Supplementary Fig. 1**c**, **d**).

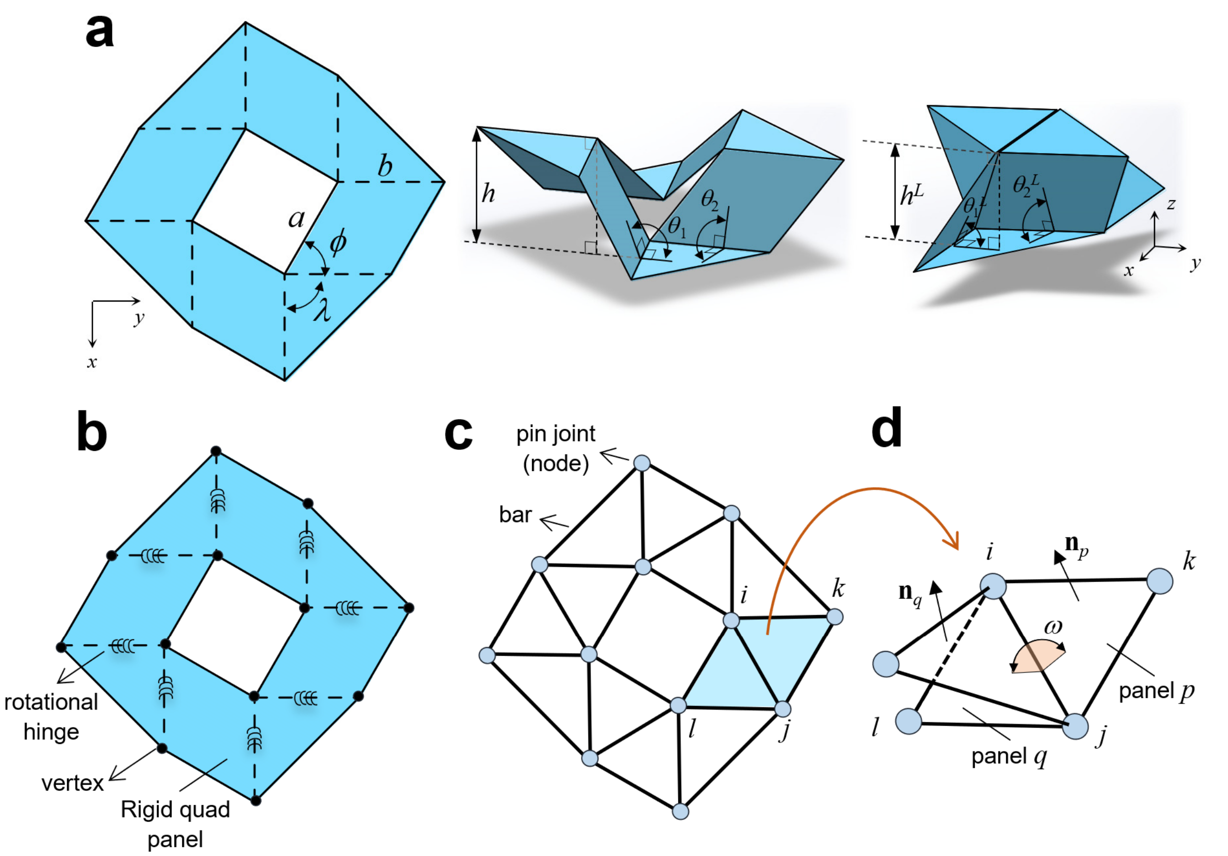

Supplementary Fig. 1 Unit chain with its representative bar and hinge kinematic model. a Geometric parameters describing our reconfigurable unit with $N = 4$. Superscript L assigned to a given variable in the first locked configuration mode of the unit chain, when all dihedral angels θ_i are equal. **b** Equivalent network of rigid panels connected through flexible hinges replaced by **c** a bar and pin-joint mechanism described by parameters used to impose planarity condition **d**.

To determine the first set of constraints for panel rigidity, we first denote the position of joint i by vector $\mathbf{x}_i$ (boldface used here for vectors and tensors) and joint j by vector $\mathbf{x}_j$; the bar connecting joint i to joint j is given by the vector $\mathbf{r}_{ij} = \pm(\mathbf{x}_i - \mathbf{x}_j)$ with length $l_{ij} = \|\mathbf{r}_{ij}\|$ where $\| \quad \|$ represents the vector norm. With this notation, the first-order inextensibility condition for bar $\mathbf{r}_{ij}$ can be written by equating the first derivative of the bar length l_{ij} to zero, $dl_{ij} = 0$, as

$$\mathbf{r}_{ij} \cdot \left(d\mathbf{x}_i - d\mathbf{x}_j\right) = 0 \tag{S1}$$

where $d\mathbf{x}_i$ and $d\mathbf{x}_j$ are the infinitesimal displacements of the joints i and j, respectively.

The second set of constraints imposes panel planarity for the quad faces, to impede the rotation of triangular faces that make the quad panel. It is formulated as follows. Assume a quad panel connecting joints i, j, k, and l, and comprising two triangular panels p (connecting joints i, j and k) and q (connecting joints i, j and l), with the normal vectors $\mathbf{n}_p = \mathbf{r}_{ij} \times \mathbf{r}_{ik}$ and $\mathbf{n}_q = \mathbf{r}_{ij} \times \mathbf{r}_{il}$ (Supplementary Fig. 1**d**). The angle between two adjacent panels p and q can be expressed as $\omega = \cos^{-1}\frac{\mathbf{n}_p \cdot \mathbf{n}_q}{\|\mathbf{n}_p\|\|\mathbf{n}_q\|}$. Equating the first differential of the angle ω to zero, $d\omega = 0$, gives the coplanarity condition for the quad panel as

$$\frac{\partial\omega}{\partial\mathbf{x}_i} d\mathbf{x}_i + \frac{\partial\omega}{\partial\mathbf{x}_j} d\mathbf{x}_j + \frac{\partial\omega}{\partial\mathbf{x}_k} d\mathbf{x}_k + \frac{\partial\omega}{\partial\mathbf{x}_l} d\mathbf{x}_l = 0. \tag{S2}$$

The rigidity constraints, Eqs. (S1) and (S2), can be expressed in matrix form for all panels as

$$\mathbf{R} \cdot d\mathbf{x} = \begin{bmatrix}\mathbf{B}\\ \mathbf{P}\end{bmatrix} \cdot d\mathbf{x} = 0 \tag{S3}$$

where $\mathbf{R}$ is the rigidity matrix consisting of the inextensibility constraints (compatibility) matrix $\mathbf{B}$ and the planarity constraints matrix $\mathbf{P}$, and $d\mathbf{x}$ is the infinitesimal displacement vector of the joints.
The relations above can now be used to determine the number of degrees of freedom (DoF) m, of the pin-jointed triangulated network [1] as

$$m = dn_j - n_K - r \tag{S4}$$

where n_K is the number of external kinematic constraints, such as those confining the rigid motions, and r is the rank of the rigidity matrix $\mathbf{R}$ in Eq. (S3). In Eq. (S4), d represents the dimensions of the problem; for spatial mechanisms d is 3. For an infinitesimally rigid structure m is zero, while for infinitesimal mechanisms $m > 0$.

With the rigidity matrix, we can now use the singular value decomposition (SVD) to determine the kinematic paths that our unit can travel upon bifurcation and track its configuration using a predictor-corrector type incremental method [2].

The SVD of the rigidity matrix $\mathbf{R} = \mathbf{W}\mathbf{V}^T\mathbf{U}^T$ is composed of a set of left singular values matrices $\mathbf{U} = [\mathbf{U}_r \vdots \mathbf{U}_m]$ and right singular values matrices $\mathbf{W} = [\mathbf{W}_r \vdots \mathbf{W}_s]$[1], and a set of non-zero singular values matrix

$$\mathbf{V} = \begin{bmatrix} \mathrm{diag}(v_1, \dots, v_r) & 0 \\ 0 & 0 \end{bmatrix}. \tag{S5}$$

$\mathbf{U}$ is a block matrix comprising the extensional deformation submatrix $\mathbf{U}_r = [\bar{\mathbf{u}}_1, \dots, \bar{\mathbf{u}}_r]$ and the orthogonal sets of m inextensional deformation (mechanism) vectors $\mathbf{U}_m = [\mathbf{u}_1, \dots, \mathbf{u}_m]$. $\mathbf{W}$ comprises a kinematically compatible extension matrix $\mathbf{W}_r = [\bar{\mathbf{w}}_1, \dots, \bar{\mathbf{w}}_r]$ and the orthogonal sets of s kinematically incompatible extension (states of self-stress) vectors $\mathbf{W}_s = [\mathbf{w}_1, \dots, \mathbf{w}_s]$, [2].

Prior to bifurcation, our unit stands in a configuration defined by the vector $\mathbf{C}^i$ and can move along one path defined by the dihedral angle relation $\theta_1 = \theta_2 = \dots = \theta_m$. Post-bifurcation can follow multiple paths, each specified by its own configuration $\mathbf{C}^{i'}$. To find $\mathbf{C}^{i'}$, we introduce a displacement amplitude parameter δ, and impose a small displacement to $\mathbf{C}^i$ by adding a finite amplitude of its inextensional deformation $\mathbf{u}_j^i$, previously obtained from the first-order SVD. This leads to a new set of configurations

$$\mathbf{C}^{i'} = \mathbf{C}^i + \delta \mathbf{u}_j^i \mathrm{sign}(\mathbf{u}_j^{i-1}.\mathbf{u}_j^i) \tag{S6}$$

where $\mathrm{sign}(\mathbf{u}_j^{i-1}.\mathbf{u}_j^i)$ ensures that the sign of δ controls the reconfiguration direction, as SVD does not guarantee the direction of the inextensional deformation $\mathbf{u}_j^i$ [3].

When we use the linear perturbation equation, Eq. (S6), to obtain the updated configuration, the length of the inextensible bars does not remain constant. To eliminate the excess elongation, $\mathbf{e}$, of the bars imposed by Eq. (S6), we iteratively find the nearest strain-free configuration by adding a correcting nodal displacement vector ($\mathbf{d}$), [3]

$$\mathbf{d} = -\sum_{j=1}^{r} \frac{\bar{\mathbf{w}}_j^T \mathbf{e}}{v_j} \bar{\mathbf{u}}_j, \tag{S7}$$

to the predicted configuration $\mathbf{C}^{i'}$ as

$$\mathbf{C}^{i+1} = \mathbf{C}^{i'} + \mathbf{d}. \tag{S8}$$

[1] s denotes the number of states of self-stress (redundant constraints present in the structure) and can be calculated using $s = n_b + n_P - r$, in which n_P is the number of planarity constraints.

When a new path has been found, we must control the independency of the kinematic paths. We do so by comparing the newly found path with other paths. This strategy is necessary since using the predictor-corrector algorithm with the first-order analysis does not guarantee that the obtained path converges to a distinct finite mechanism. The predictor-corrector algorithm is implemented in MATLAB, and all the simulations are based on an in-house developed code.

The algorithm can execute large displacement simulations, but cannot find all possible post-bifurcation kinematic paths. We resort to principles of *Pólya Enumeration Theory* that enables us to find all independent post-bifurcation kinematic paths, as explained in Supplementary Discussion S3.

S2. Additional constraints (imposed by unit chain stacking)

A single unit kinematic chain has multiple DoFs that lead to certain configurations, and the fully developed (flat) configuration is one among others. To reduce its DoF we resort to layer stacking, which leads our unit to share certain panels with those of the units placed above and below. Supplementary Fig. 2**a** shows a physical prototype where only the triangular panels are shared between two units, whereas the others are not shared. Here, the non-zero thickness of adjacent hinged panels causes an offset between the hinges. Supplementary Fig. 2**b** shows the only possible rigid body motion the planes can undergo. The quad planes cannot rotate in the same direction, shown in Supplementary Fig. 2**c**. This is due to the internal forces exerted by planes and hinges. The outcome is that both plane interference and hinge offset geometrically constraint the unit to respect the rigid body motion. For example, the motion in Supplementary Fig. 2**c** is only possible if the quad planes can undergo flexural deformation (Supplementary Fig. 2**d**), which violates our rigid motion assumption.

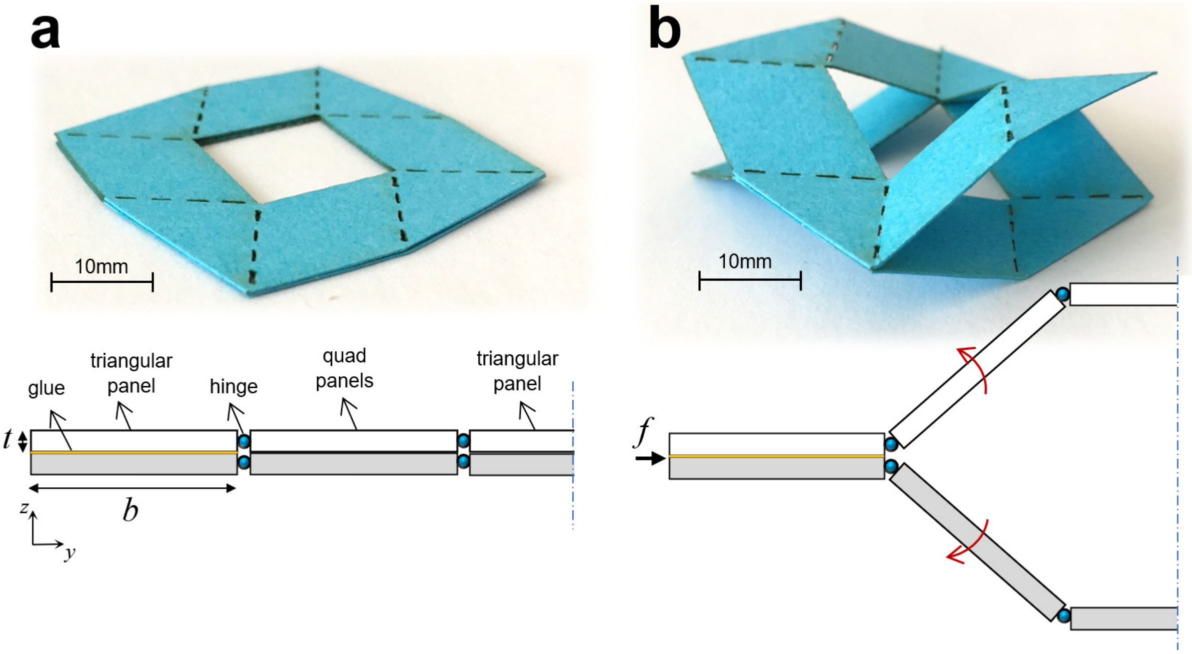

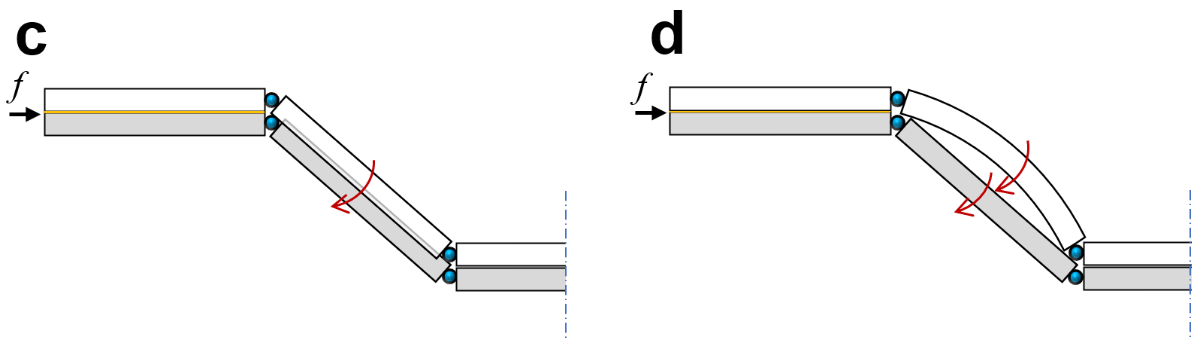

Supplementary Fig. 2 Layer stacking as a pathway to reduced mobility. a Flat configuration for a two-layered unit kinematic chain $\widehat{\mathbf{N}}_{\mathbf{4}}\mathbf{n}_{\mathbf{2}}$ made of non-negligible thickness panels. **b** A kinematic path of the multilayered units, where two adjacent quad panels rotate in opposite directions under in-plane load f. **c** Example of inadmissible kinematic path for two quad panels rotating in identical direction under in-plane load f; this kinematic path violates the rigidity assumption of the panels and is feasible only if the neighboring panels can deform **d**.

S3. Pólya enumeration theorem

The post-bifurcation kinematic analysis given in the previous section cannot guarantee that all post-bifurcation paths are found [3]. We now use principles of *Pólya Enumeration Theory* [4] to identify and count all independent post-bifurcation kinematic paths and available modes. The Pólya Enumeration Theorem is instrumental to identify the reconfiguration modes of our unit kinematic chain.

As stated in the main text of the paper, the problem of finding all independent *regular* modes of $\mathrm{N}_N\mathrm{n}_n$ is equivalent to the classical necklace problem in combinatorics. There, the goal is to find the number of necklace arrangements we can construct from knowledge of $N/2$ colored beads, each painted in two distinct colors, e.g., white or black. In our case, the dihedral angle $\boldsymbol{\mathcal{A}}$ of our unit chain represents one color, e.g., white, and $\boldsymbol{\mathcal{O}}$ the other, e.g., black. Due to the mountain and valley assignment, the transformations are defined on hypothetical $N/2$ sided polygons since congruent triangular faces are located on every other corner of the original chain. Thus, the necklace problem of an N even-sided polygonal unit chain, N_N, with constraints of mountains and valleys degenerates to an $N/2$ sided necklace.

We start by stretching the necklace with $\frac{N}{2}$ beads into the shape of a uniform $\frac{N}{2}$-sided regular polygon with one bead at each corner. We define the set of the vertices of this polygon by $S = \{\chi_1, \chi_2, \chi_3, \ldots, \chi_{\frac{N}{2}}\}$ and the set of binary colors by $R = \{\boldsymbol{\mathcal{A}}, \boldsymbol{\mathcal{O}}\}$, and introduce set X = {all possible coloring arrangement of S by R}. Next, we define rigid body transformations of the necklace. We assume that the beads can rotate around the necklace, and the necklace can be flipped over. These operations are considered as *rigid motions* applied to the necklace.

Supplementary Fig. 3 depicts the set of all rigid body motions, each N_N unit chain with N = 4, 6, 8 and 10 can undergo. We denote them with the set $G = \{\pi_1, \pi_2, \pi_3, \ldots, \pi_N\}$, where π_j $(j = 1, \ldots, N)$ are the members of the dihedral symmetry group, $D_{N/2}$, for a uniform $N/2$-sided regular polygon that acts on the set, X, of the necklace problem. The members of G are defined as

$$\pi_i \in G|\, i \leq \frac{N}{2}:\ \pi_i = \mathrm{Rot}_z\left((i-1)\frac{4\pi}{N}\right)$$
$$\pi_i \in G|\, i > \frac{N}{2}:\ \pi_i = \mathrm{Ref}_{a_i} \quad \text{(S9)}$$

where $\mathrm{Rot}_z(\alpha)$ is the rotation operator about the z axis through the angle α, and Ref_{a_i} is a reflection operator about the axis a_i whose angles with the x axis can be obtained from

$$\text{If } \tfrac{N}{2} \in \mathbb{E} : \beta_i = (i-1)\tfrac{2\pi}{N} \quad \text{for} \quad i \leq N \text{ , and}$$
$$\text{If } \tfrac{N}{2} \in \mathbb{O} : \beta_i = (i-1)\tfrac{4\pi}{N} \quad \text{for} \quad i \leq \tfrac{N}{2}. \quad \text{(S10)}$$

As each rigid motion permutes the elements in X and S, we can represent these rigid motions by their permutations on S or X. For each N, there are exactly N rigid body transformations, N/2 rotation operations and N/2 reflection operations (Supplementary Fig. 3). We note that for N_4, two rigid body motions are equivalent.

Supplementary Fig. 3 shows both the original unit kinematic chain and its necklace counterpart at the top of each table for a given N. In each case, the dark and light shadings of the kinematic unit chain (left) denote the mountain- and valley-faces, respectively. In the necklace representation (right), each number specifies a corresponding pair of valley dihedral angles. For example, N_4 has two pairs of valley-dihedral angle and thus its equivalent necklace has 2 beads, shown as ① and ②. Below the first row of each kinematic chain, the possible rigid motion operations, rotation and reflection, are shown. In particular, the successive application of rigid body motions makes the vertices alternate and form a periodic loop, namely a cycle. A cycle is described by a number, or a series of numbers, placed in one bracket underneath each kinematic unit chain. The length of a cycle is the number of elements in each bracket. A string of cycles, i.e., a sequence of brackets, describes a rigid motion.

To illustrate the above, we consider N_6 as an example for the case of 0˚ rotation. We note the vertex assignment is identical to the initial assignment. The transformation corresponding to its rigid motion is the identity operation, which maps every vertex to itself regardless of the number of times the transformation is applied. Its vertex counterpart can be written as (1)(2)(3). By using the *Pólya's enumeration theorem,* this rigid motion is made of three cycles each of length one. On the other hand, a 120˚ rotation applied to N_6 is described by (132), which consists of 1 cycle of length three.

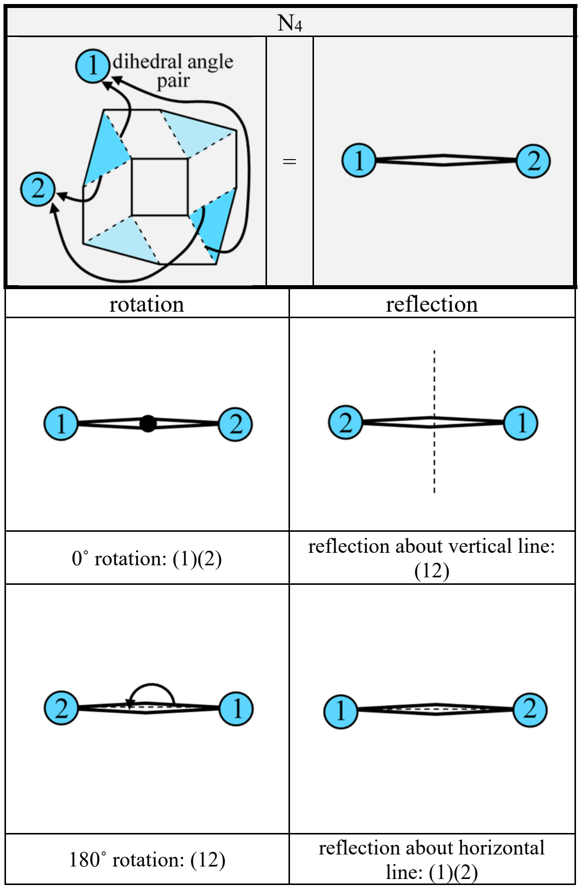
N4
dihedral angle pair
1
2
=
1
2
rotation
reflection
0˚ rotation: (1)(2)
reflection about vertical line: (12)
180˚ rotation: (12)
reflection about horizontal line: (1)(2)

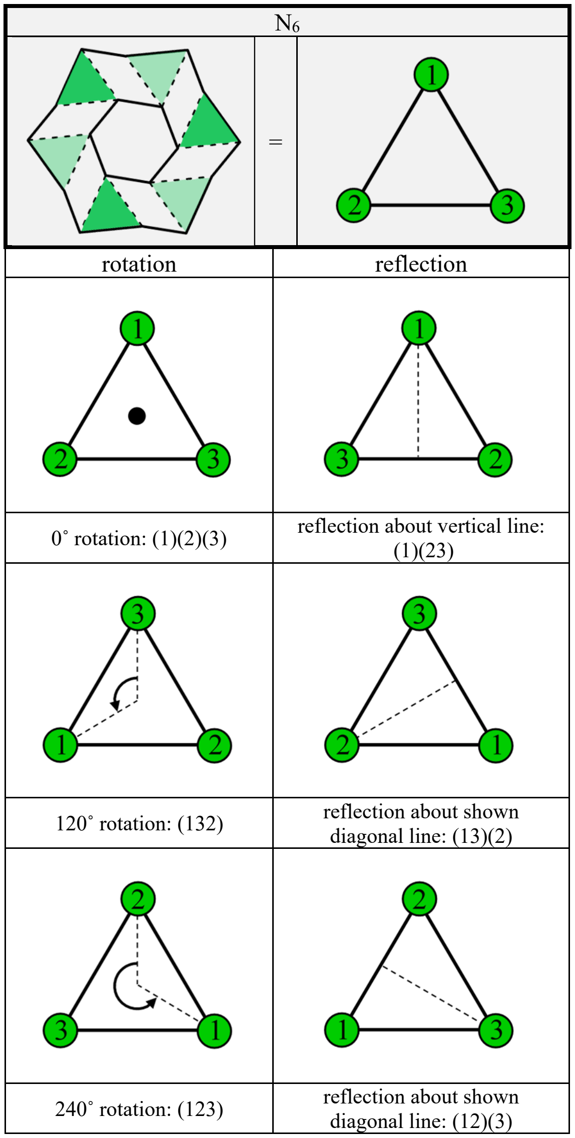
N6
=
1
2
3
rotation
reflection
0˚ rotation: (1)(2)(3)
reflection about vertical line: (1)(23)
120˚ rotation: (132)
reflection about shown diagonal line: (13)(2)
240˚ rotation: (123)
reflection about shown diagonal line: (12)(3)

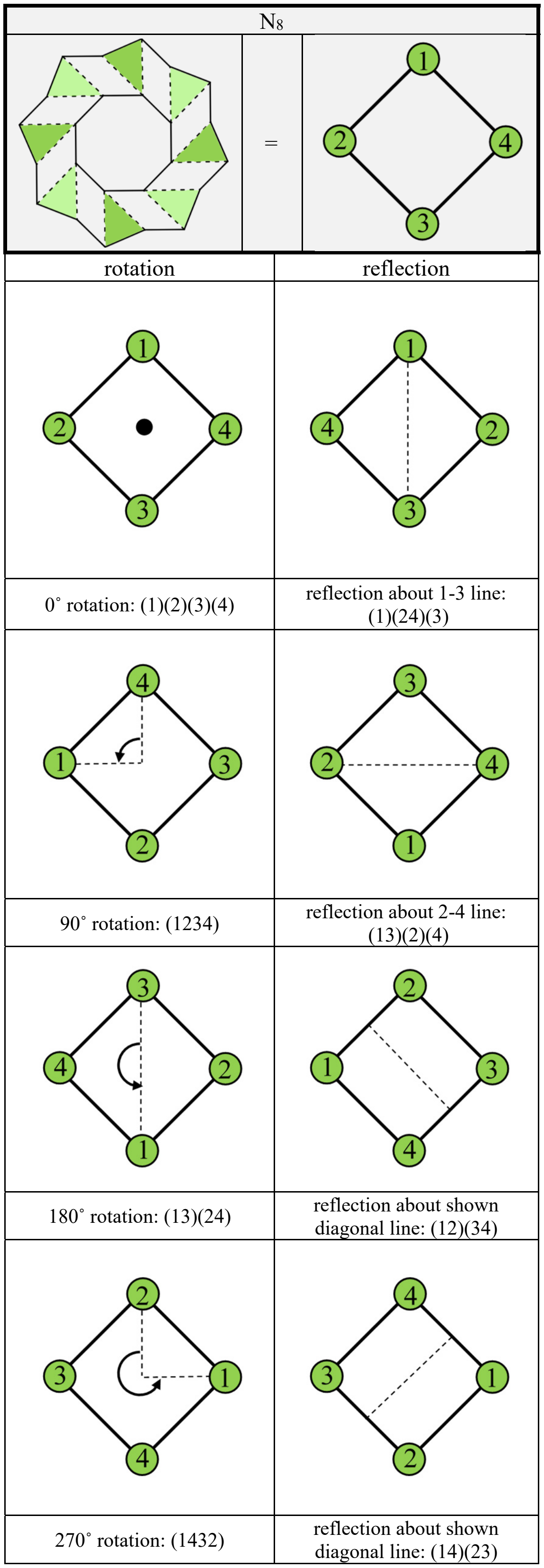
N8
rotation
reflection
0˚ rotation: (1)(2)(3)(4)
reflection about 1-3 line: (1)(24)(3)
90˚ rotation: (1234)
reflection about 2-4 line: (13)(2)(4)
180˚ rotation: (13)(24)
reflection about shown diagonal line: (12)(34)
270˚ rotation: (1432)
reflection about shown diagonal line: (14)(23)

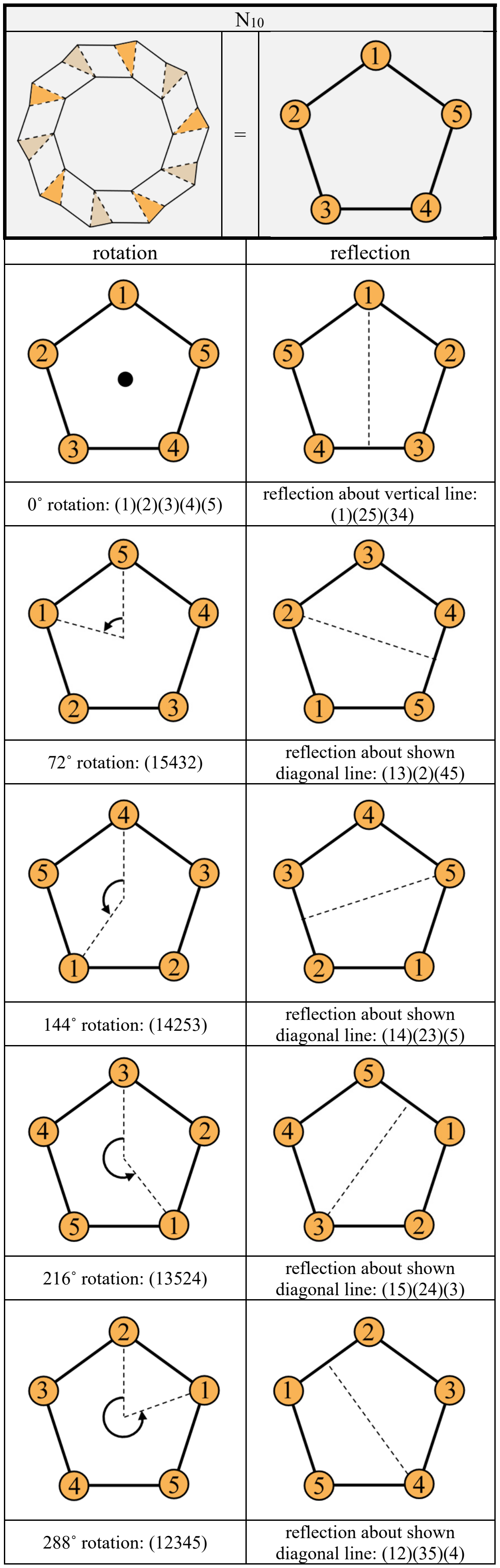
N10
rotation
reflection
0˚ rotation: (1)(2)(3)(4)(5)
reflection about vertical line: (1)(25)(34)
72˚ rotation: (15432)
reflection about shown diagonal line: (13)(2)(45)
144˚ rotation: (14253)
reflection about shown diagonal line: (14)(23)(5)
216˚ rotation: (13524)
reflection about shown diagonal line: (15)(24)(3)
288˚ rotation: (12345)
reflection about shown diagonal line: (12)(35)(4)

Supplementary Fig. 3 Schematic representation of the unit kinematic chains with its equivalent necklace. Schematic representation of unit kinematic chain with N = 4, 6, 8 and 10 (left) and its equivalent necklace (right) with all its possible rigid motions below the first row for each unit chain. Two valley "dihedral angle pairs" are shown as an example for N_4 only. Symmetries of the necklaces are also represented as permutation of the vertices.

Below the first row are the possible rigid body motions each unit chain can undergo. The alternating light and dark colors on the triangular faces of the kinematic chain refer to mountain and valley assignments, respectively. The equivalent necklace has numbered vertices and describes a given rigid motion. Below each rigid motion is a sequence of parentheses describing the rigid body motion in cycle notation. A black dot represents zero-degree rotation. A combination of dashed lines and circular arcs represent the rotation operations. A dashed line is used for reflection operations and indicates the mirror line.

Assume $\pi_j \in G$ is a rigid motion operation that can be described by a combination of its cycles. Now we can assign a monomial to every π_j. The monomial is the product of $\chi_{i_1}, \chi_{i_2}, \ldots, \chi_{i_\tau}$ where $i_1, i_2, \ldots i_\tau$ represent the lengths of the cycles in the rigid operation π_j. The monomial can be written as $f_{\pi_j} = \chi_{i_1}\chi_{i_2} \cdots \chi_{i_\tau}$, and the cycle index $f(\chi_1, \chi_2, \ldots, \chi_w)$ of G acting on S can be written as the sum of the monomials

$$f(\chi_1, \chi_2, \ldots, \chi_w) = \frac{1}{|G|} \sum_{\pi_j \in G} f_{\pi_j} \qquad \text{(S11)}$$

where w is the length of the longest cycle in the vertex representation of each of $\pi_j \in G$ in Supplementary Fig. 3. The number of distinct patterns in X under the corresponding action of G on X is $f(\varrho, \varrho, \ldots, \varrho)$, where ϱ is the length of the set R, $\varrho = |R|$; in our case $\varrho = 2$.

If we denote the general set of color arrangements by $R = \{\mathcal{C}_1, \mathcal{C}_2, \ldots, \mathcal{C}_\tau\}$, the pattern inventory of X can be defined as $f(\mathcal{C}_1 + \mathcal{C}_2 + \cdots + \mathcal{C}_\tau, {\mathcal{C}_1}^2 + {\mathcal{C}_2}^2 + \cdots + {\mathcal{C}_\tau}^2, \ldots, {\mathcal{C}_1}^w + {\mathcal{C}_2}^w + \cdots + {\mathcal{C}_\tau}^w)$. The Pólya Enumeration Theorem states that if $\kappa \mathcal{C}_1^{i_1} \mathcal{C}_2^{i_2} \ldots \mathcal{C}_\tau^{i_\tau}$ appears in the pattern inventory of X, then there are κ patterns in X where $\mathcal{C}_1$ appears i_1 times, $\mathcal{C}_2$ appears i_2 times, …, and $\mathcal{C}_\tau$ appears i_τ times. The summation of the coefficients κ of the pattern inventory gives the number of necklace arrangements. For example, the N_8n_n unit is analogous to a necklace with four beads where $R = \{\boldsymbol{\mathcal{A}}, \boldsymbol{\mathcal{O}}\}$, and its pattern inventory becomes $f(\boldsymbol{\mathcal{A}} + \boldsymbol{\mathcal{O}}, \boldsymbol{\mathcal{A}}^2 + \boldsymbol{\mathcal{O}}^2, \boldsymbol{\mathcal{A}}^3 + \boldsymbol{\mathcal{O}}^3, \boldsymbol{\mathcal{A}}^4 + \boldsymbol{\mathcal{O}}^4) = \boldsymbol{\mathcal{A}}^4 + \boldsymbol{\mathcal{A}}^3\boldsymbol{\mathcal{O}} + 2\boldsymbol{\mathcal{A}}^2\boldsymbol{\mathcal{O}}^2 + \boldsymbol{\mathcal{A}}\boldsymbol{\mathcal{O}}^3 + \boldsymbol{\mathcal{O}}^4$. The mode is lockable when at least two successive acute dihedral angles $\boldsymbol{\mathcal{A}}$ exist in the pattern sequence. For example, in the case of N_8n_n, the lockable mechanisms are $\boldsymbol{\mathcal{A}}^2\boldsymbol{\mathcal{O}}^2$ (i.e., the one with the sequence of $\boldsymbol{\mathcal{A}\mathcal{A}\mathcal{O}\mathcal{O}}$), $\boldsymbol{\mathcal{A}}^3\boldsymbol{\mathcal{O}}$ and $\boldsymbol{\mathcal{A}}^4$.

Supplementary Table 1 Lockable and flat-foldable modes. Top view of the nearly developed, locked, and flat-folded states of $N_N n_n$ unit kinematic chains for N = 4, 6, 8 and 10. Light orange shading indicates flat-foldability and light blue refers to lockability. The number of modes per given N is reported in the last row.

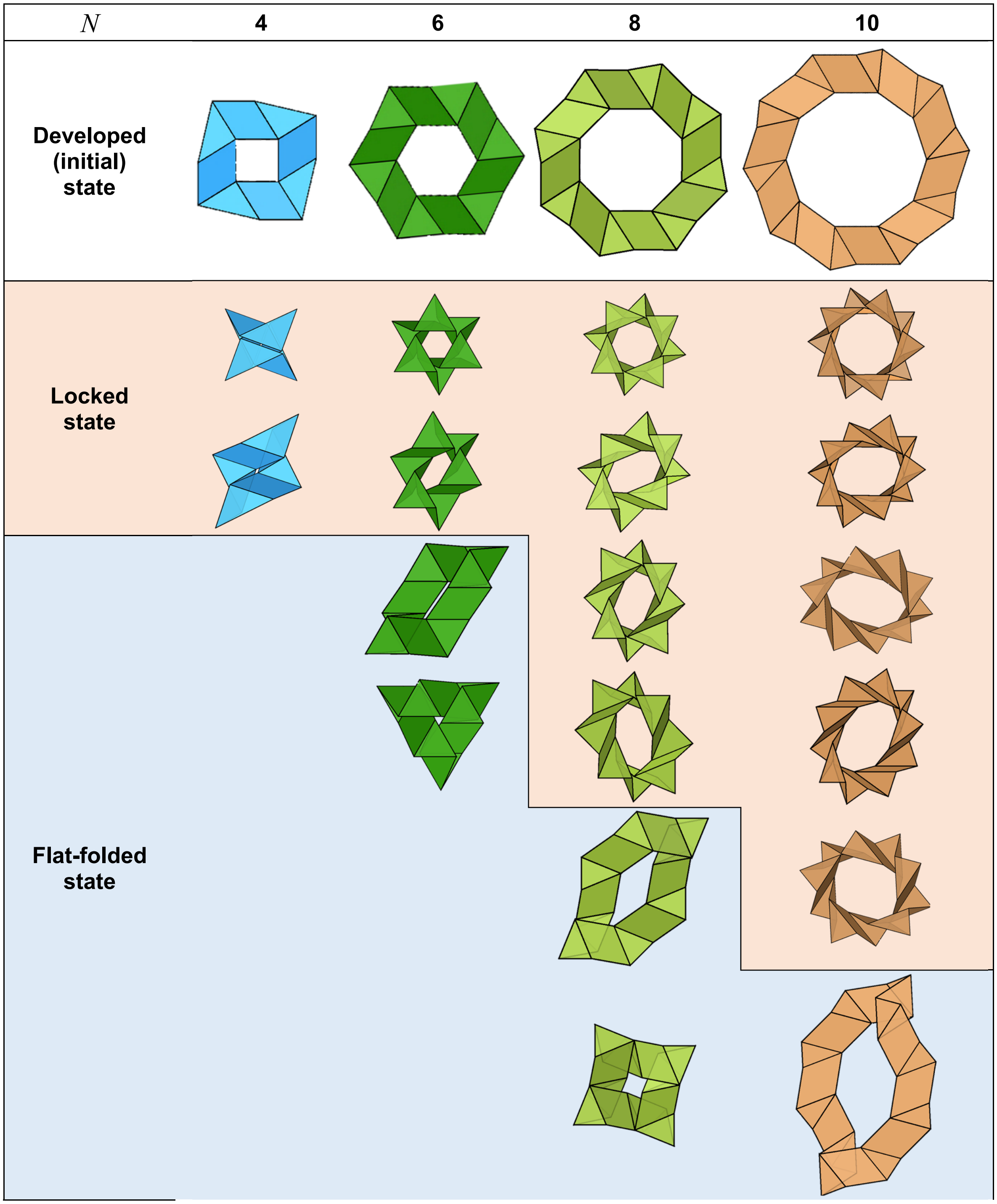

				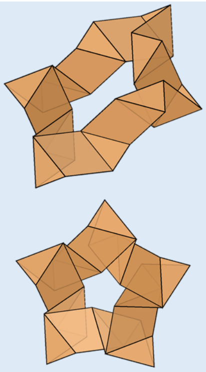
**# of modes (N-2)**	**2**	**4**	**6**	**8**

S4. Tessellations: from individual stacks of unit chains to a periodic cellular material

Each multilayered unit chain can be joined with others to form a periodic material system that can reconfigure along either lockable or flat-foldable modes. To examine their in-plane tessellation patterns (see Supplementary Fig. 4**b**), we refer to the periodic array our $\mathrm{N}_N\mathrm{n}_n$ forms in 2 dimensions, with centroids falling onto a simple Bravais lattice of the *base-centered orthorhombic* family (Supplementary Fig. 4**a**).

Here, we study ways of connecting multilayered unit chains to tile the x-y plane while preserving the characteristic kinematics. We do so by joining in-plane the triangular rigid panels of our multilayered unit chains, without making use of flexible hinges at their interface. This strategy is realized through two types of connections: 1) the whole triangular panel is shared between two multilayered unit chains, i.e., the two patterns overlap (see for example the tessellation pattern for N = 6 in Supplementary Fig. 1**b**); 2) the interface of the base edge of their triangular panels acts as connection; this means that the two triangular panels form a single parallelogram panel that is rigid (see for example, the tessellation pattern for N = 4 in Supplementary Fig. 1**b**). This leads us to conceive two classes of tessellation containing similar unit chains:

i) Most packed tessellation patterns. This class describes the densest lattices that can be made from our primitive units,

ii) Less packed tessellation patterns. This class includes realizations of other possible low-density lattices; it is analyzed through the notion of macro-chains.

Other types of connection between dissimilar units are possible, but are not examined here.

S4.1. Most packed tessellation patterns

The most compact form of tessellation can be described by a periodic array of $\mathrm{N}_N\mathrm{n}_n$ multilayered unit chains in two dimensions, whose centroids (centers of mass) fall onto a simple Bravais lattice of the *base-centered orthorhombic* family with basis vectors {$\mathbf{e}_1$, $\mathbf{e}_2$}.

Let us consider a rhombus constructed from vertices that are the centroids of the four adjacent unit chains in a tessellated pattern (Supplementary Fig. 4**a**). The geometric constraints imply that its vertex angle $\gamma \equiv \cos^{-1}(\mathbf{e}_1 \cdot \mathbf{e}_2) \geq \frac{\pi}{3}$ impedes the overlap of unit chains along the short diagonal of the rhombus (Supplementary Fig. 4**a**). Additionally, the vertex angle γ must be made of ζ segments of λ (where $\lambda = \frac{2\pi}{N}$), i.e., $\gamma = \zeta\lambda = \zeta\frac{2\pi}{N}$, and its supplementary angle γ' must be made of ζ' segments of λ, i.e., $\gamma' = \zeta'\lambda$. All admissible angles γ can be obtained by solving the linear Diophantine equation [5]

$$\zeta + \zeta' = N/2 \tag{S12}$$

which is written assuming the condition $\gamma + \gamma' = \pi$ (see Supplementary Fig. 4**a**).

Supplementary Table 2 shows all possible solutions (ζ , ζ') of Eq. (S12) for a given N (representing the unit kinematic chain $\mathrm{N}_N\mathrm{n}_n$), with the resulting vertex angle γ ($= \zeta\frac{2\pi}{N}$). The choice of the smallest ζ among both solutions (if two solutions exist) gives the most packed pattern. For example, for N =16 we have two possible solutions of Eq. (S12): (ζ , ζ') = (3,5) and (4,4), from which we calculate the resultant vertex angle γ as 67.50˚ and 90˚. The former gives the most packed tessellation pattern. On the other hand, if $\gamma = \frac{\pi}{3}$, the tessellation belongs to the *hexagonal* family, while $\gamma = \frac{\pi}{2}$ results in a tessellation of the *tetragonal* family.

Supplementary Table 2 Most packed tessellation parameters. All possible solutions for Eq. (S12), i.e., (ζ , ζ'), and resultant acute rhombus angles γ for the most compact tessellation patterns of $\mathrm{N}_N\mathrm{n}_n$ for N = 4 to 22.

N	Number of solutions of Eq. (S12)	Possible solutions of (ζ , ζ')	Angle of rhombus $\gamma = \zeta\lambda$
4	1	(1,1)	90˚
6	1	(1,2)	60˚
8	1	(2,2)	90˚
10	1	(2,3)	72˚
12	2	(3,3), (2,4)	90˚, 60˚
14	1	(3,4)	77.14˚
16	2	(3,5), (4,4)	67.50˚, 90˚
18	2	(3,6), (4,5)	60˚, 100˚
20	2	(5,5), (4,6)	90˚, 72˚
22	2	(4,7), (5,6)	65.45˚, 81.82˚

Unit kinematic chains can connect along the periodic base vectors, $\mathbf{e}_1$ and $\mathbf{e}_2$, through the base of the isosceles triangulated panels (Supplementary Fig. 4). In certain cases, e.g., *hexagonal* family ($\gamma = \frac{\pi}{3}$) with an odd ζ (see for example, the case N = 18 in Supplementary Table 3), the base of the isosceles triangulated panels coincides, and an extra connection along the short diagonal of the rhombus can be considered. The bonds in the most packed tessellation patterns of $\mathrm{N}_4\mathrm{n}_n$ and $\mathrm{N}_6\mathrm{n}_n$ are a shared parallelogram panel intersecting the basis vectors $\mathbf{e}_1$ and $\mathbf{e}_2$.

S4.2. Less-packed tessellation patterns using macro-chains

An alternative way to tessellate our pattern is to start from a macro-chain containing primitive units. A macro-chain is constructed by the in-plane connection of a certain number of similar $\mathrm{N}_N\mathrm{n}_n$ unit chains, i.e., units with prescribed N, to form a regular polygon; this is done by replacing each edge with one primitive unit, e.g., Supplementary Fig. 4**c**, **d**.

The shape of macro-chains is governed by the geometry of their constituents. Their existence can be formulated as follows. We denote the ith positive factor of N with $\mathcal{M}_i$. For any given $\mathcal{M}_i > 4$ one $\mathcal{M}_i$-sided regular polygonal macro-chain ($\mathrm{N}_N^{\mathcal{M}_i}\mathrm{n}_{\bar{n}}$) exists provided either of $\mathcal{M}_i$ or $\frac{N}{2\mathcal{M}_i}(\mathcal{M}_i - 2)$ becomes an even number. $\mathrm{N}_N^{\mathcal{M}_i}\mathrm{n}_{\bar{n}}$ is a macro-chain connecting the centroids of the unit chains $\mathrm{N}_N\,\mathrm{n}_{\bar{n}}$.

In-plane tessellations of macro-chains can be formulated using the method described above if they fall into one of the Bravais lattice families[2]. Supplementary Fig. 4**b** illustrates the smallest macro-chain, i.e., $\mathrm{N}_{16}^{4}\mathrm{n}_{\bar{n}}$, and its tessellation that can be built using the unit kinematic chain $\mathrm{N}_{16}\mathrm{n}_{\bar{n}}$. Supplementary Fig. 4**c**, **d**, **e** shows examples of possible tessellations one can imagine using the macro-chain of $\mathrm{N}_{16}^{8}\mathrm{n}_{\bar{n}}$; several more exist that are beyond the scope of this work. In the figures, orange lines indicate the connectivity between units in a macro-chain, and red lines describe the connectivity of macro-chains in the tessellation.

In the tessellations of $\mathrm{N}_N^{\mathcal{M}_i}\mathrm{n}_{\bar{n}}$ macro-chains, the connection of the macro-chains may be much more complex than that of the single unit chains, as inter-chain connections can also be established, e.g., red dash-lines in Supplementary Fig. 4**c**. Here, the main bonds between macro-chains occur either through the bases of the isosceles triangulated panels that intersect the direction vectors $\mathbf{e}_1$ and $\mathbf{e}_2$ (see Supplementary Fig. 4**c**, **d**), or through the overlapped unit-chains $\mathrm{N}_N\mathrm{n}_{\bar{n}}$ as shown in Supplementary Fig. 4**e**.

The results of the analysis above exemplify the breadth of the design space that can be achieved through the selection of alternative tessellation patterns. Each tessellation pattern is characterized by its own set of rigid foldability attributes, physical properties, and load-bearing capacity.

[2] $\mathcal{M}_i = 4$ results in the Bravais lattice of the tetragonal family and $\mathcal{M}_i = 3$ results in the Bravais lattice of the hexagonal family.

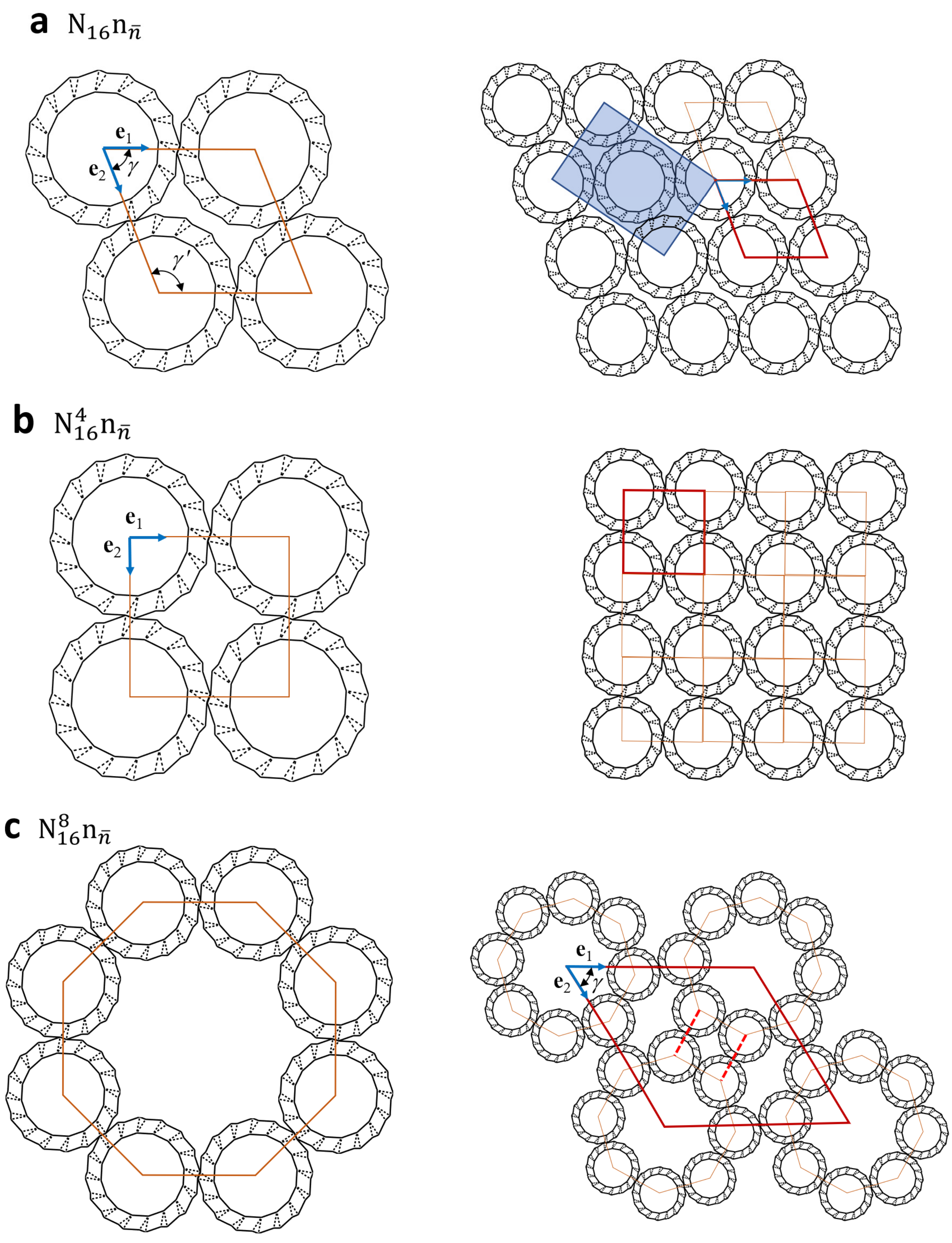
a $N_{16}n_{\bar{n}}$
e_1
e_2
γ
γ'
b $N_{16}^{4}n_{\bar{n}}$
e_1
e_2
c $N_{16}^{8}n_{\bar{n}}$
e_1
e_2
γ

d $\mathrm{N}_{16}^{8}\mathrm{n}_{\bar{n}}$

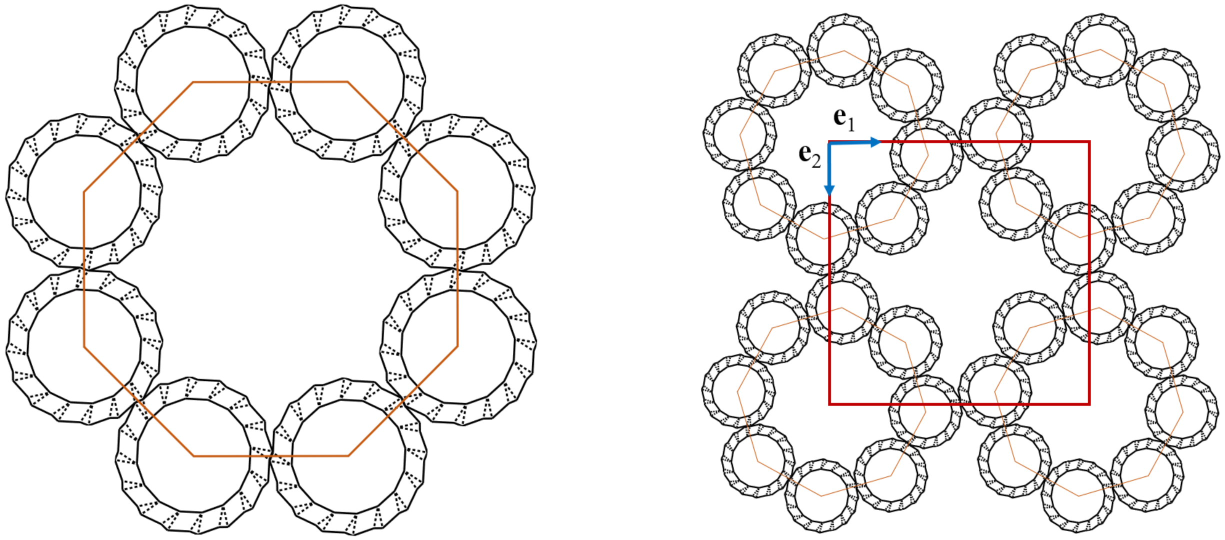

e $\mathrm{N}_{16}^{8}\mathrm{n}_{\bar{n}}$

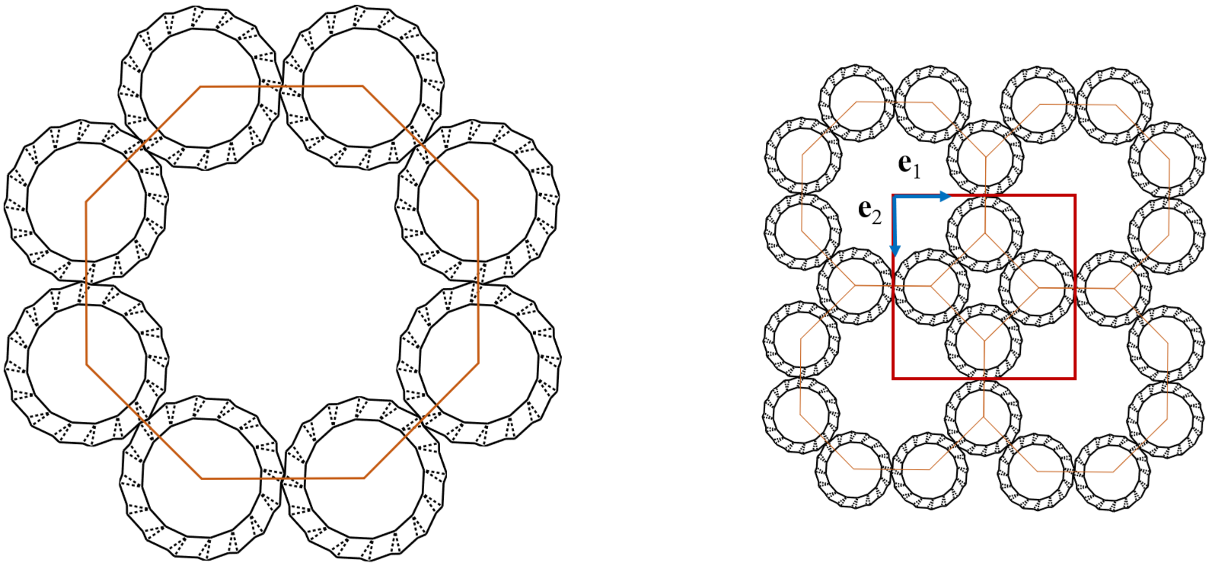

Supplementary Fig. 4 Most-packed and less-packed tessellations. In-plane view of most compact tessellations for $\mathrm{N}_{16}\mathrm{n}_{n}$ along with three other possible less-packed tessellations for $\mathrm{N}_{16}^{8}\mathrm{n}_{n}$.

S5. Rigidity

To study the mobility of our unit, we replace the actual panels with their bar and hinge counterparts. *Upon locking*, certain bars and hinges coincide (Supplementary Fig. 5). This holds only if, at the lock state, we assume there is full contact between edges and faces, and thus relevant bars and hinges condense into a single bar and a single hinge. Such an assumption underpins our rigidity analysis and is considered valid if the direction of the force is unchanged [6].

To analyze the rigidity of our unit chains one panel is constrained to eliminate rigid body motions. Supplementary Table 3 shows the number of bars and hinges in the fully developed and $\mathcal{A}^{\frac{N}{2}}$ locked states for a single layer of a given unit. For example, for N = 4, the number of bars upon locking is reduced from 36 to 30, while the joints of four pairs coincide. The reduction in the number of joints and bars bring about an increase in connectivity, which in turn leads to rigidity. For certain patterns, a certain number of layers (last row) are required to achieve rigidity. The analysis indicates that other locked configurations presented in Supplementary Table 1 can also satisfy the rigidity condition (i.e., m = 0 in Eq. (S4)).

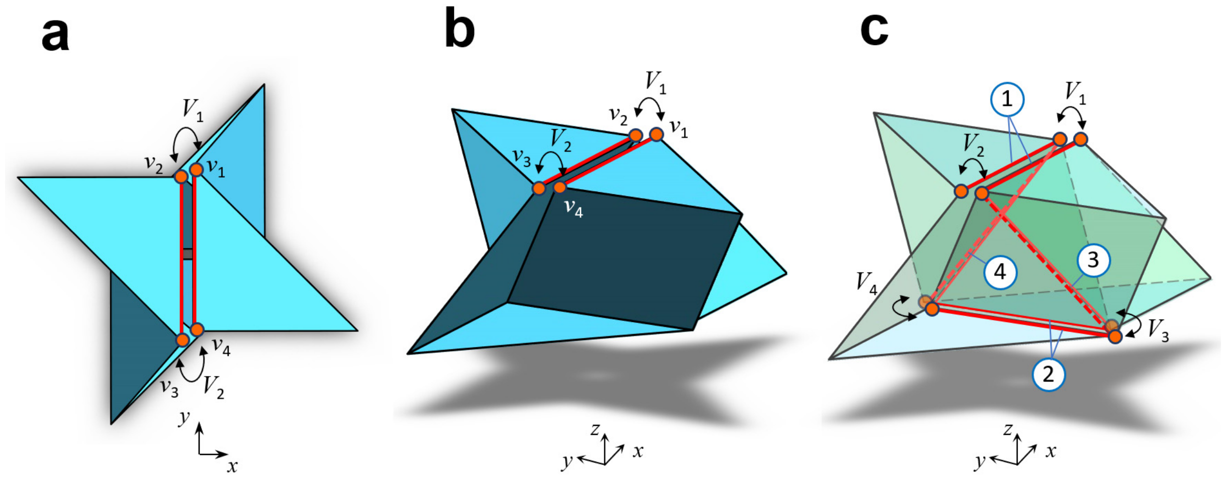

Supplementary Fig. 5 Description of topology change upon locking. Schematic of N_4n_1 unit kinematic chain showing alignment of joints and bars upon locking. **a** Top view and **b** isometric view immediately prior to locking, in **a** and **b** the coinciding points are (v_1, v_2) and (v_3, v_4), concisely shown by the pairs V_1 and V_2 respectively. **c** Isometric view showing all bars and hinges before reaching full contact, thus condensing into one element. Vertex pairs are shown by V_i; numbers in circles denote coinciding lines/edges.

Supplementary Table 3 Rigidity under compression. Bar and joint counts of $\mathrm{N}_N\mathrm{n}_n$ unit kinematic chains (top row) for N = 4, 6, 8, 10 and 12 during folding (light pink), and at the locked state (light blue), along with the number of layers to attain rigidity (dark blue). Red solid and shaded red circles represent vertices laying respectively on mountain and valley planes.

**Pattern**	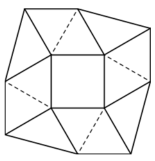	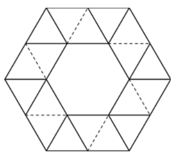	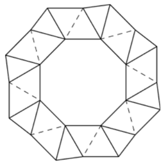	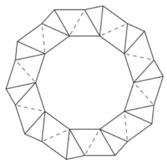	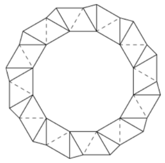
**Joints** (during folding)	12	18	24	30	36
**Bars** (during folding)	24	36	48	60	72
**Joints** (in locked state)	8	12	16	20	24
**Bars** (in locked state)	18	30	40	50	60
Top View	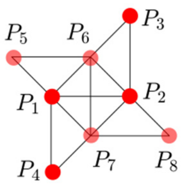	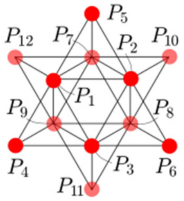	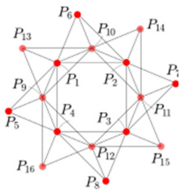	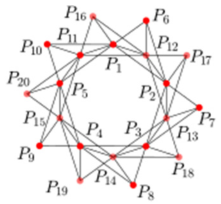	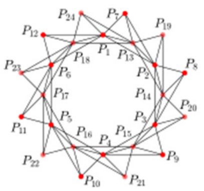
Required layers for rigidity	1	2	3	3	3

S6. Geometric mechanics of representative unit cell (RUC)

During reconfiguration, our unit chains pass a kinematic bifurcation instant that provides access to dissimilar kinematic paths, each having modes that can be either flat-foldable and lockable. Each path has its own physical characteristics, e.g., geometry, relative density, and Poisson's ratio. In this section, we first derive the closed-form expressions that describe changes in properties upon folding for $\mathrm{N}_4\mathrm{n}_n$ and $\mathrm{N}_6\mathrm{n}_n$ units. Next, we derive the representative energy landscape relationships for a given lockable and flat-foldable modes, and generate energy-phase diagram that can explain the underlying physics of activation through in-plane confinement.

S6.1 RUC model with boundary and loading conditions for N_4n_n unit cell

We examine a representative unit cell for $\mathrm{N}_4\mathrm{n}_n$ that can describe the behavior of its entire periodic tessellation. Supplementary Fig. 6 shows the RUC with its own geometric parameters and loading conditions in a Cartesian coordinate system.

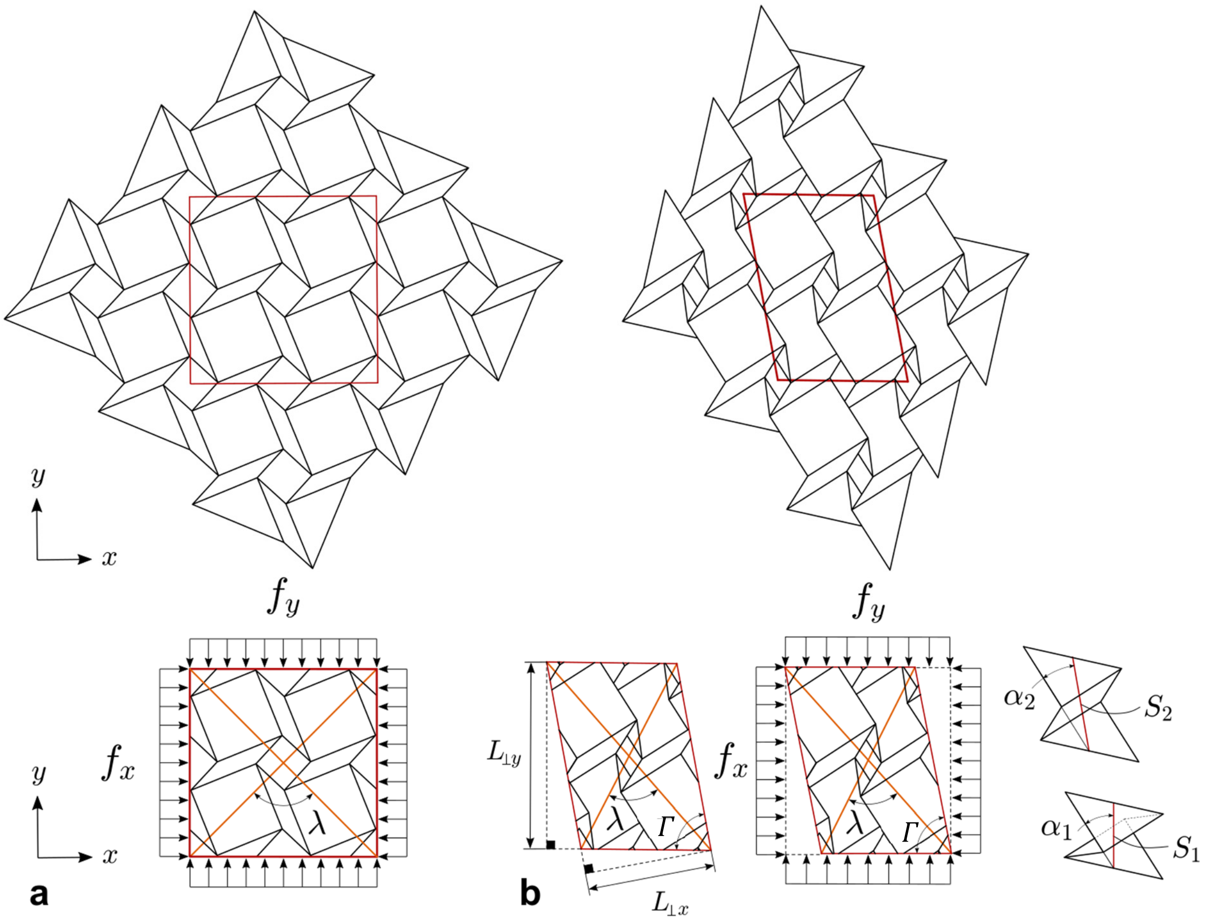

Supplementary Fig. 6 Geometry of RUC for N4 pattern. Top view of the most compact tessellations of $\mathrm{N}_4\mathrm{n}_n$ under two locking modes **a** $\mathcal{A}^2$ (or $\mathcal{O}^2$), **b** $\mathcal{AO}$, and geometric parameters used in the calculation of the total energy. RUC shown in red squares for each mode.

In modes $\mathcal{O}^2$ and $\mathcal{A}^2$, the RUC is a square (red) with side $L_1 = 2S_1$, in which S_1 is the length of the line connecting the mid-points of the bases of two adjacent triangles separated by a parallelogram (right of Supplementary Fig. 6**b**) and can be calculated as

$$S_1^2 = (a\cos\phi)^2 + (b - a\sin\phi\cos\theta)^2. \tag{S13}$$

Upon kinematic bifurcation, the square shape of the RUC switches to a parallelogram. One of its sides can be computed from Eq. (S13), while the other side $L_2 = 2S_2$ can be calculated by replacing $\pi - \theta$ in Eq. (S13) as

$$S_2^2 = (a\cos\phi)^2 + (b + a\sin\phi\cos\theta)^2. \tag{S14}$$

To obtain the skewness angle of the parallelogram angle Γ in Supplementary Fig. 6**b**, we first calculate the angles α_1 and α_2 from

$$\alpha_1 = \tan^{-1}\left(\frac{a\cos\phi}{b - a\sin\phi\cos\theta}\right)$$
$$\alpha_2 = \tan^{-1}\left(\frac{a\cos\phi}{b + a\sin\phi\cos\theta}\right) \quad \text{(S15)}$$

and then, replace them in the relation

$$\Gamma = 90^\circ - \alpha_1 + \alpha_2. \quad \text{(S16)}$$

Then, the cross-section area of the RUC in the x-y plane can be obtained from

$$A_{RUC} = L_1 L_2 \sin\Gamma \quad \text{(S17)}$$

S6.2 RUC model with boundary and loading conditions for $N_6 n_n$ unit

We now examine $\mathrm{N}_6\mathrm{n}_n$ unit and derive geometric relations that describe the deformations of its kinematic paths. In this case, the smallest unit with parallel edges is a rectangle. The length of the line connecting the midpoints of the two adjacent triangles are

$$S_1^2 = (a\cos\phi)^2 + \left(\frac{2h}{3} - a\sin\phi\cos\theta\right)^2. \quad \text{(S18)}$$

In $\mathcal{O}^3$ and $\mathcal{A}^3$ modes, the sides of the RUC shown in Supplementary Fig. 7 are given by

$$L_x = 3S_1 \quad \text{(S19)}$$
$$L_y = \sqrt{3}S_1$$

A kinematic bifurcation can bring the unit into $\mathcal{A}^2\mathcal{O}$ or $\mathcal{O}^2\mathcal{A}$ modes. The new length of the line connecting the midpoints of the two adjacent triangles, S_2, can be obtained by replacing $\pi - \theta$ in Eq. (S17) as

$$S_2^2 = (a\cos\phi)^2 + \left(\frac{2h}{3} + a\sin\phi\cos\theta\right)^2. \quad \text{(S20)}$$

The angles α_1 and α_2 representing the inclination of the S_1 and S_2 lines with respect to the vertical axis can be obtained as

$$\alpha_1 = \tan^{-1}\left(\frac{a\cos\phi}{\frac{2h}{3} - a\sin\phi\cos\theta}\right) \text{ and } \alpha_2 = \tan^{-1}\left(\frac{a\cos\phi}{\frac{2h}{3} + a\sin\phi\cos\theta}\right). \quad \text{(S21)}$$

After bifurcation, the hexagons that are defined by the centroids of the triangular faces are distorted. The internal angles ψ_i can be determined as a function of the inclination angles $\alpha_1$and α_2 as

$$\psi_1 = 120^\circ + \alpha_1 - \alpha_2,$$
$$\psi_2 = 240^\circ - \psi_1, \quad \text{(S22)}$$
$$\psi_3 = 120^\circ.$$

If we connect the centroids of every other triangle around a unit chain, we obtain an additional triangle. The sides of this triangle relates to S_1 and S_2, and ψ_is through

$$L_1^2 = S_1^2 + S_2^2 - 2S_1S_2\cos\psi_1,$$
$$L_2^2 = S_1^2 + S_2^2 - 2S_1S_2\cos\psi_2, \quad \text{(S23)}$$

$L_3 = S_1\sqrt{3}$.

The parallelogram of the RUC is defined by two triangles. One of the sides of the parallelogram is L_1 while the other side is given by

$$L_1'^{\,2} = 2L_1^2 + 2L_3^2 - L_2^2. \tag{S24}$$

Now, using the cosine rule, we obtain

$$\lambda_2 = \cos^{-1}\left(\frac{L_1^2+L_3^2-L_2^2}{2L_1L_3}\right). \tag{S25}$$

The angle of the parallelogram can be obtained from

$$\Gamma = \lambda_2 + \lambda_2' \tag{S26}$$

where λ_2' can be calculated using the sine rule as

$$\frac{L_1'}{\sin\lambda_1} = \frac{L_1}{\sin\lambda_2'} \ . \tag{S27}$$

Then, the cross-section area of the RUC in the *x-y* plane can be obtained from

$$A_{RUC} = L_1L_1'\sin\Gamma. \tag{S28}$$

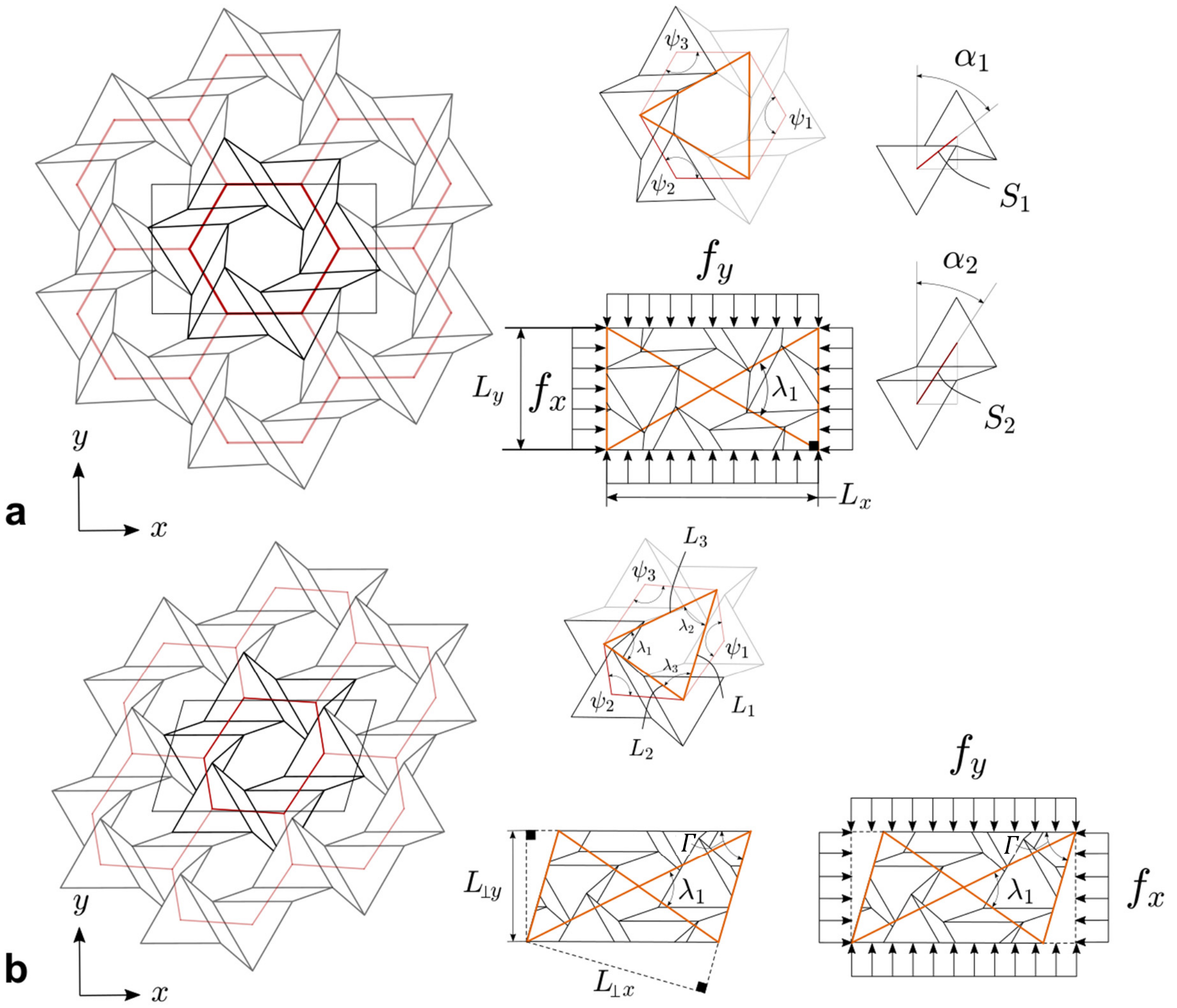

Supplementary Fig. 7 Geometry of RUC for N₆ pattern. Top view of the most compact tessellations of the $\mathrm{N}_6\mathrm{n}_n$ under two locking modes **a** $\mathcal{A}^3$ and **b** $\mathcal{A}^2\mathcal{O}$ along with their geometrical parameters used in the calculation of the energy of the hinges (Supplementary Discussion S9.2). RUC shown in black squares for each mode.

S7. Relative density

The relative density of our multimodal rigid-foldable materials upon reconfiguration (and transition between modes) can be expressed as a function of the dihedral angle θ and other geometric parameters of the unit chain for a given tessellation type. In addition, upon kinematic bifurcation multiple paths emerge, and a distinct relative density can be associated to each given kinematic path. Supplementary Fig. 8 shows the relevant geometric parameters for $\mathrm{N}_{10}\mathrm{n}_n$, here taken as example for the analysis.

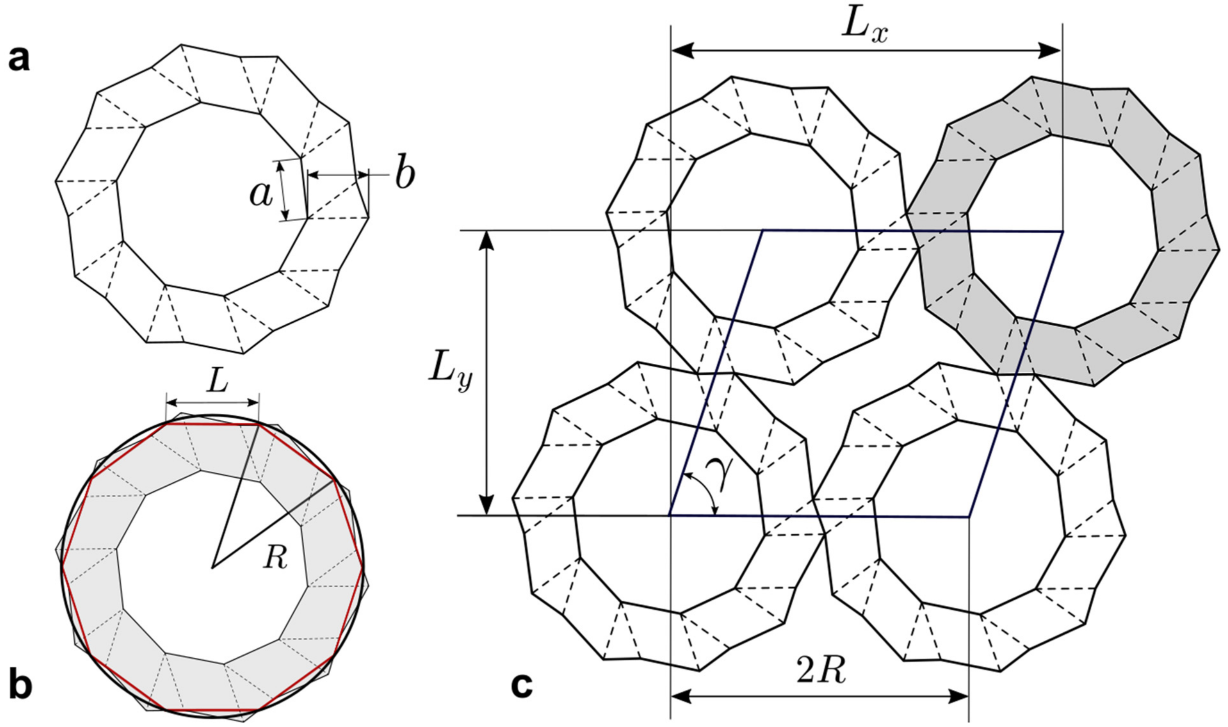

Supplementary Fig. 8 General geometric parameters of our tessellated units. a Geometric parameters of a unit kinematic chain; **b** Connecting the mid-point of the bases of the triangles yields an N-sided polygon, shown in red; **c** Geometric parameters of the Bravais lattice tessellation. Regardless of N, four units can always be combined to form a rhombus defined by the angle γ, which is obtained from Eq. (S12).

Supplementary Fig. 8**b** shows that by connecting the mid-points of the bases of the triangles, we obtain a regular polygon (red decagon here). R is the radius of the circumscribing circle of the polygon. The side of the polygon, L, is given by

$$L = \sqrt{(a\cos\phi)^2 + (b\sin\lambda - a\sin\phi\cos\phi)^2}, \tag{S29}$$

which relates to R through

$$L = 2R\sin\frac{\lambda}{2}. \tag{S30}$$

The volume of the rhombus shown in Supplementary Fig. 8 with height h (recall that it is now a 3D structure) for modes $\boldsymbol{\mathcal{O}}^{N/2}$ and $\boldsymbol{\mathcal{A}}^{N/2}$ can be related to R using

$$V_f = 4aR^2\sin\theta\sin\phi\sin\gamma. \tag{S31}$$

The volume of the solid material inside the rhombus is

$$V_s = Nt\left(\frac{1}{2}b^2\sin\lambda + ab\sin\phi\right). \tag{S32}$$

Therefore, the relative density can be expressed as

$$\bar{\rho} = \frac{V_s}{V_f} = \frac{Nt\left(\frac{1}{2}b^2\sin\lambda + ab\sin\phi\right)}{4aR^2\sin\theta\sin\phi\sin\gamma}. \tag{S33}$$

Supplementary Fig. 9 shows the relative density calculated for the $\widehat{\mathrm{N}}_4\mathrm{n}_n$ and $\widehat{\mathrm{N}}_6\mathrm{n}_n$ lattices when they reconfigure through their two kinematic paths. While Eq. (S33) can be used to obtain the relative density of $\widehat{\mathrm{N}}_N\mathrm{n}_{\bar{n}}$ units in their uniform reconfiguration (for modes $\boldsymbol{\mathcal{O}}^{N/2}$ and $\boldsymbol{\mathcal{A}}^{N/2}$), we resort to Supplementary Figs. 6 and 7 for the parameters defining the RUC to calculate the relative density of other kinematic paths. The volume of the solid material within the RUC can still be obtained from Eq. (S31). The volume of the RUC can be obtained by multiplying the height by the area of the cross-section of the RUC in the *x-y* plane. We observe the relative density of flat-foldable modes approaches infinity as the lattice approaches its flat-foldable configuration. Also, each deformation mode attains a certain minimum relative density during the reconfiguration process. As we expect, lock mode $\boldsymbol{\mathcal{A}}^{N/2}$ has the highest density of all locked configurations, while $\boldsymbol{\mathcal{O}}^{N/2}$ is the least dense flat-foldable mode.

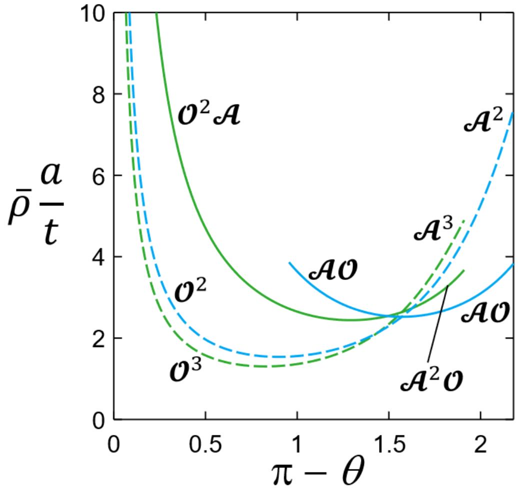

Supplementary Fig. 9 Relative density upon reconfiguration for different lock and flat-fold modes. Relative density computed for $\widehat{\mathrm{N}}_4\mathrm{n}_n$ and $\widehat{\mathrm{N}}_6\mathrm{n}_n$ lattices during reconfiguration along their two kinematic paths. t is the panel thickness, and a is the length of the sides of the primitive regular polygon.

S8. Poisson's ratio

During folding our metamaterials exhibit auxetic in-plane behavior. The following derives expressions of the Poisson's ratio that emerge at given modes.

S8.1. Poisson's ratio of $\mathcal{A}^{N/2}$ and $\mathcal{O}^{N/2}$ modes

To obtain the expressions of the Poisson's ratio for $\widehat{\mathrm{N}}_n\mathrm{n}_n$, we begin by tessellating the rhombus of Supplementary Fig. 8. By doing so, we can express the increments of the strains, i.e., $d\varepsilon_x$, $d\varepsilon_y$ and $d\varepsilon_z$, in terms of the derivatives of the parameters $\mathcal{L}_x$, $\mathcal{L}_y$ and the height of the $\widehat{\mathrm{N}}_n\mathrm{n}_n$ unit. We first express $\mathcal{L}_x$, $\mathcal{L}_y$ as a function of the radius of the circumscribing circle, R, and the skewness angle γ.

$$\begin{aligned} \mathcal{L}_x &= 2R(1+\cos\gamma) \\ \mathcal{L}_y &= 2R\sin\gamma \end{aligned} \tag{S34}$$

ν_{xy}, the Poisson's ratio in x-y plane, can be written as the ratio of the increments of the in-plane strains

$$\nu_{xy} = -\frac{d\varepsilon_y}{d\varepsilon_x} = -\frac{{d\mathcal{L}_x}/{\mathcal{L}_x}}{{d\mathcal{L}_y}/{\mathcal{L}_y}} = -1. \tag{S35}$$

This value remains constant during the folding process.

ν_{xz} and ν_{yz}, the out-of-plane Poisson's ratios, can be obtained from

$$\nu_{xz} = \nu_{yz} = -R\cot\theta\frac{d\theta}{dR} \tag{S36}$$

Taking the derivative of Eqs. (S34) and substituting the derivatives of R and θ from Eqs. (S29) and (S30) into Eq. (S36), we obtain

$$\nu_{xz} = \nu_{yz} = \frac{((a\cos\phi)^2 + (b\sin\lambda - a\sin\phi\cos\theta)^2)\cos\theta}{a(b\sin\lambda - a\sin\lambda\cos\theta)\sin\phi\sin^2\theta} \tag{S37}$$

Interestingly, we observe a change in the sign of the out of plane Poisson's ratios. This entails that passing the kinematic bifurcation, our folding metamaterials in $\boldsymbol{\mathcal{A}}^{N/2}$ mode have omnidirectional auxetic behavior.

S8.2. Poisson's ratio of other modes

The Poisson's ratio in other modes depends on the lattice (i.e., $\widehat{\mathrm{N}}_N\mathrm{n}_n$) and the kinematic path. We examine here $\widehat{\mathrm{N}}_4\mathrm{n}_n$ and $\widehat{\mathrm{N}}_6\mathrm{n}_n$ and present the results of the Poisson's ratio for other mode shapes.

To simplify the calculation, we refer to the RUC in Supplementary Figs. 6 and 7. In this case, the in-plane Poisson's ratio expression can be written as

$$\nu_{xy} = -\frac{d\varepsilon_y}{d\varepsilon_x} = -\frac{d\mathcal{L}_y/\mathcal{L}_y}{d\mathcal{L}_x/\mathcal{L}_x} = -\frac{dL_\perp/L_\perp}{dL_1/L_1} = -\frac{d(L_2\sin\Gamma)/L_2\sin\Gamma}{dL_1/L_1} \tag{S38}$$

where the parameters L_1, L_2 and Γ are presented in Eq. (S13), Eq. (S14) and Eq. (S16) for N_4 pattern. The expression for the Poisson's ratio of N_6 pattern can be written as

$$\nu_{xy} = -\frac{d\varepsilon_y}{d\varepsilon_x} = -\frac{d\mathcal{L}_y/\mathcal{L}_y}{d\mathcal{L}_x/\mathcal{L}_x} = -\frac{dL_\perp/L_\perp}{dL_1'/L_1'} = -\frac{d(L_2\sin\Gamma)/L_2\sin\Gamma}{dL_1'/L_1'} \tag{S39}$$

The parameter L_2, L_1' and Γ can be obtained from Eqs. (S23), (S24) and (S26). Supplementary Fig. 10 shows the in-plane Poisson's ratio calculated for $\widehat{\mathrm{N}}_4\mathrm{n}_n$ and $\widehat{\mathrm{N}}_6\mathrm{n}_n$ evolving along their kinematic paths. We observe that the in-plane Poisson's ratio of each lattice highly depends on the tessellation and kinematic path as opposed to that of the modes $\boldsymbol{\mathcal{A}}^{N/2}$and $\boldsymbol{\mathcal{O}}^{N/2}$.

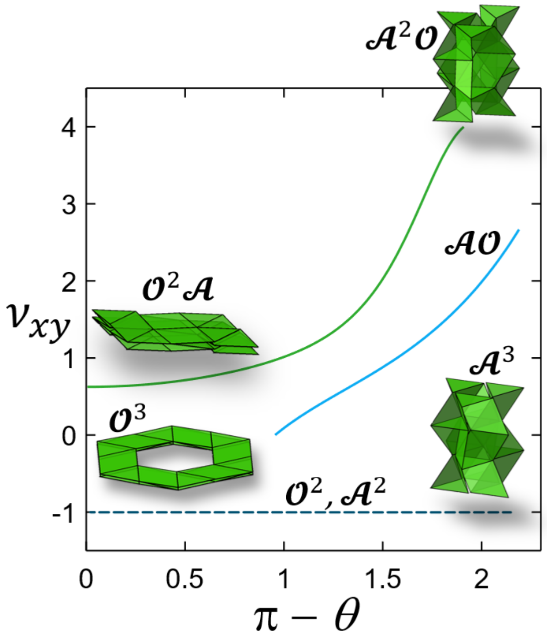

Supplementary Fig. 10 Poisson's ratio upon reconfiguration for different lock and flat-fold modes. Poisson's ratio calculated for the $\widehat{\mathrm{N}}_4\mathrm{n}_n$ and $\widehat{\mathrm{N}}_6\mathrm{n}_n$ lattices along their kinematic paths.

S9. Energy analysis

Here we derive the energy expressions for $\mathrm{N}_4\mathrm{n}_n$ and $\mathrm{N}_6\mathrm{n}_n$ as a function of their geometric parameters. Our analysis assumes the rigid panels are hinged with linear rotational springs, which represent the compliance of finite-thickness ligaments connecting panels. We also assume that our hinges maintain a linear behavior for most of the folding process.

S9.1. Energy expression of $\boldsymbol{N_N n_n}$ under in-plane biaxial loading

We focus on the energy expressions of $\mathrm{N}_N\mathrm{n}_n$ under in-plane loading. The expressions are derived with reference to Supplementary Figs. 6 and 7. The total potential energy of RUC can be written as

$$\Pi = \mathcal{n}_{chain/RUC}\left(\sum_{i=1}^{\xi} 2nkb(\theta-\theta^0)^2 + \sum_{i=1}^{\xi'} 2nkb(\pi-\theta-\theta^0)^2\right) - \int_{L_{\perp x0}}^{L_{\perp x}} f_x d\ell - \int_{L_{\perp y0}}^{L_{\perp y}} f_y d\ell \tag{S40}$$

where ξ is the total number of pairs for the valley acute dihedral angles ($\boldsymbol{\mathcal{A}}$) and ξ' counts the total number of pairs for the valley obtuse dihedral angle ($\boldsymbol{\mathcal{O}}$) in a unit chain with $\xi + \xi' = \frac{N}{2}$, and $\mathcal{n}_{chain/RUC}$ is the number of unit chains within a single RUC. For example, for the RUC of N_4 pattern this value is $\mathcal{n}_{chain/RUC} = 2$ and for the RUC of N_6 pattern $\mathcal{n}_{chain/RUC} = 1$ (see Supplementary Figs. 6 and 7). n is the number of stacked layers, k the stiffness per unit length of the hinge, b the extrusion length, θ and θ^0 are the current and initial (zero-energy configuration) dihedral angles respectively. i indicates the pair of parallelograms with identical

dihedral angle, f_x and f_y are the in-plane forces. $L_{\perp_x}$ and $L_{\perp_y}$ are the distances of the boundaries of RUC where f_x and f_y are applied (Supplementary Fig. 6), and can be defined as

$$\begin{aligned} L_{\perp_x} &= L_x \sin\Gamma \\ L_{\perp_y} &= L_y \sin\Gamma \end{aligned} \tag{S41}$$

Moreover, the initial distances of the RUC boundaries are $L_{\perp_{x0}}$ and $L_{\perp_{y0}}$. Eq. (S40) is valid for all in-plane loading conditions of $\mathrm{N}_N\mathrm{n}_n$.

S9.2. Energy analysis of $\boldsymbol{N_4 n_n}$ under in-plane biaxial loading

Starting from a flat configuration (i.e., $\xi = 0,\ \theta^0 = 0$) and applying biaxial loads, we can find a closed form expression of the critical bucking load that is necessary to move from a flat configuration to $\boldsymbol{\mathcal{O}}^2$ mode. By taking the first variation of the energy expression with $\xi' = 0$ in the first lock mode and setting $\theta = 180°$, we obtain the critical load as

$$f_x + f_y = \frac{8nk\bar{b}}{\sin\phi}\frac{\sqrt{\left(\bar{b}+\sin\phi\right)^2+\cos^2\phi}}{\left(\bar{b}+\sin\phi\right)}. \tag{S42}$$

This boundary is shown by line I in Supplementary Fig. 11. Passing the bifurcation, $\mathrm{N}_4\mathrm{n}_n$ buckles into its first mode. A load increase makes the metamaterial to continue its deformation in the $\boldsymbol{\mathcal{O}}^2$ mode until $f_x + f_y$ reaches the following limit, which corresponds to the kinematic bifurcation at $\theta = 90°$ (line II in Supplementary Fig. 11)

$$f_x + f_y = \frac{4nk\pi}{\sin\phi}\sqrt{\cos^2\phi + \bar{b}^2}. \tag{S43}$$

To determine the post-bifurcation behavior, we compare the slope of the energy for dissimilar modes at $\theta = 90°$. The path taken by our metamaterial is the one with the lowest gradient.

To obtain the boundaries of $\boldsymbol{\mathcal{AO}}$ and $\boldsymbol{\mathcal{A}^2}$ modes, i.e., lines III and IV in Supplementary Fig. 11, we note that the $\boldsymbol{\mathcal{AO}}$ mode is activated when the slope of the energy at $\theta = 90°$ is below the slope of the energy in $\boldsymbol{\mathcal{A}^2}$ mode. This condition can be written as

$$\left(\delta\Pi_{\mathcal{AO}} < \delta\Pi_{\mathcal{A}^2}\right)\Big|_{\theta=\frac{\pi}{2}} \rightarrow \left(\frac{\partial\Pi_{\mathcal{AO}}}{\partial\theta} < \frac{\partial\Pi_{\mathcal{A}^2}}{\partial\theta}\right)\Big|_{\theta=\frac{\pi}{2}} \rightarrow \left(\frac{\partial\Pi}{\partial\theta}\Big|_{\xi'=1} < \frac{\partial\Pi}{\partial\theta}\Big|_{\xi'=0}\right)\Big|_{\theta=\frac{\pi}{2}}. \tag{S44}$$

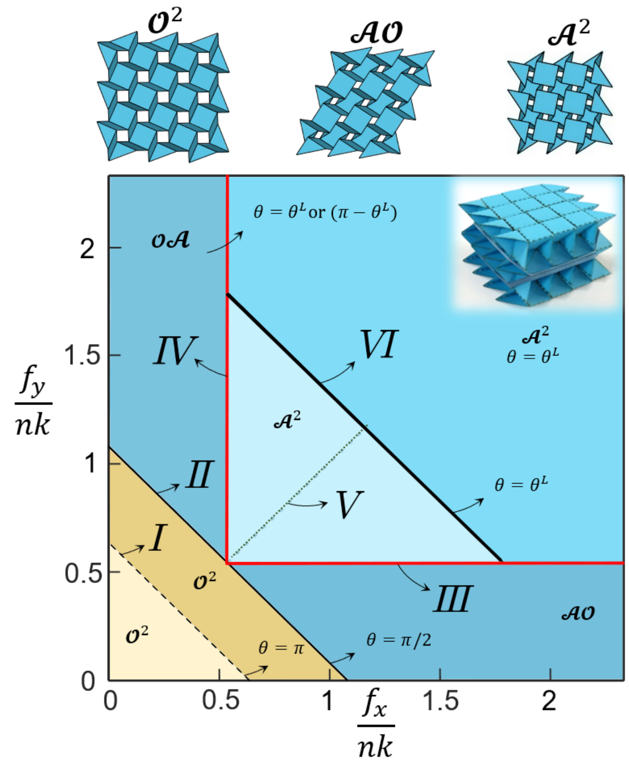

Supplementary Fig. 11 Phase diagram of $\widehat{\mathbf{N}}_4\mathbf{n}_n$ under biaxial loads.

Upon substituting the derivative of Eq. (S40) into Eq. (S44), and rearranging we obtain

$$f_x \text{ or } f_y \leq 2nk\pi \frac{\sqrt{\bar{b}^2 + \cos^2\phi}}{\sin\phi}. \tag{S45}$$

The equality signs in Eq. (S45) define the boundary of $\mathcal{AO}$ ($\mathcal{OA}$) with $\mathcal{A}^2$ (lines III and IV in Supplementary Fig. 11). If the compressive forces are equal while passing the bifurcation (line V in Supplementary Fig. 11), the metamaterial shifts to mode $\mathcal{A}^2$. The reconfiguration in mode $\mathcal{A}^2$ continues until the loads reach a limit defined by the lock angle. This limit is obtained by taking the variation of Eq. (S40) and solving for $\theta = \theta^L$ as

$$f_x + f_y = \frac{8nk\bar{b}(\pi - \theta^L)}{\sin\phi \sin\theta^L} \frac{\sqrt{\cos^2\phi + (\bar{b} - \sin\phi\cos\theta^L)^2}}{\bar{b} - \sin\phi\cos\theta^L}. \tag{S46}$$

This limit is shown by line VI in Supplementary Fig. 11.

S9.3. Energy analysis of $N_6 n_n$ under in-plane biaxial loading

To obtain the reconfiguration behavior of $\mathrm{N}_6\mathrm{n}_n$ we use Eq. (S40). The length of the sides of RUC in Supplementary Fig. 7 can be obtained by using

$$L_{\perp_x} = L_1 \sin\Gamma,$$
and (S47)
$$L_{\perp_y} = L_1' \sin\Gamma.$$

The parameters L_1, L_1', and Γ are calculated from Eqs. (S23), (S24), and (S26), respectively. In the case of N6, ξ is equal to 3 for mode $\mathcal{A}^3$, while for mode $\mathcal{A}^2\mathcal{O}$, ξ is 2.

Supplementary Fig. 12 shows the phase diagram of N6. Initially, the metamaterial is assumed to be flat. By compressing it, a certain limit is reached, and the metamaterial buckles to $\mathcal{O}^3$ (line I in Supplementary Fig. 12). This limit can be obtained by taking the first variation of Eq. (S40) at $\theta = 180°$ as

$$3f_x + \sqrt{3}f_y = \frac{12nk\bar{b}}{\sin^2\phi}\frac{\sqrt{\sin^2\phi\left(\frac{2}{3}\sin\theta+1\right)^2+\cos^2\phi}}{\left(\frac{2}{3}\sin\theta+1\right)}. \tag{S48}$$

This deformation mode is maintained until the metamaterial reaches $\theta = 90°$ (line II in Supplementary Fig. 12). The equation of line II can be obtained from the first variation of Eq. (S40) by setting $\theta = 90°$. Accordingly, this limit can be expressed as

$$3f_x + \sqrt{3}f_y = \frac{9nk\bar{b}}{\sin^2\phi\sin\theta}\sqrt{\left(\frac{2\sin\phi\sin\theta}{3}\right)^2 + \cos^2\phi}. \tag{S49}$$

This also applies to the case of N4. At the bifurcation point, the ratio of the forces plays a major role in governing the resulting deformation path. The boundaries of $\mathcal{A}^2\mathcal{O}$, $\mathcal{O}^2\mathcal{A}$, and $\mathcal{A}^3$ modes can be obtained by comparing the slope of the corresponding energy expressions. The smallest slope defines the deformation after the kinematic bifurcation. Therefore, we can write

$$\left(\delta\Pi_{\mathcal{A}^2\mathcal{O}} \le \delta\Pi_{\mathcal{A}^3}\right)\Big|_{\theta=\frac{\pi}{2}} \to \left(\frac{\partial\Pi_{\mathcal{A}^2\mathcal{O}}}{\partial\theta} \le \frac{\partial\Pi_{\mathcal{A}^3}}{\partial\theta}\right)\Big|_{\theta=\frac{\pi}{2}} \to \left(\frac{\partial\Pi}{\partial\theta}\Big|_{\xi'=2} < \frac{\partial\Pi}{\partial\theta}\Big|_{\xi'=3}\right)\Big|_{\theta=\frac{\pi}{2}}, \tag{S50}$$

which can be simplified as

$$f_x\left(\frac{2}{3} - \sqrt{3}\frac{\cot\phi}{\sin\theta}\right) + f_y\left(\frac{2}{\sqrt{3}} + \frac{\cot\phi}{\sin\theta}\right) \le 2nk\bar{b}\pi\frac{\sqrt{\left(\frac{2\sin\phi\sin\theta}{3}\right)^2+\cos^2\phi}}{\sin^2\phi\sin\theta}. \tag{S51}$$

Note that the equality sign represents line III in Supplementary Fig. 12.

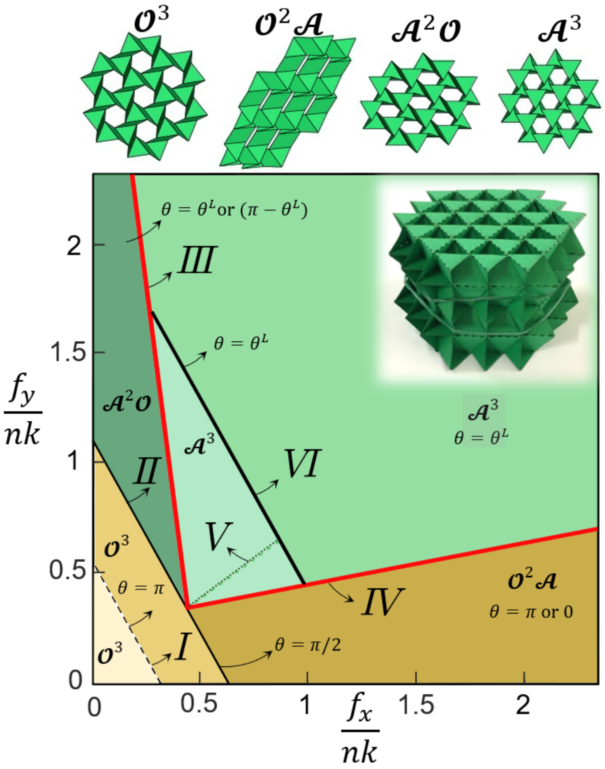

Supplementary Fig. 12 Phase diagram of $\hat{\mathrm{N}}_6\mathbf{n}_n$ under biaxial loads.

The boundary of $\mathcal{O}^2\mathcal{A}$ and $\mathcal{A}^3$ can be obtained from

$$\left(-\delta\Pi_{\mathcal{A}^3} \le \delta\Pi_{\mathcal{O}^2\mathcal{A}}\right)\Big|_{\theta=\frac{\pi}{2}} \to \left(-\frac{\partial\Pi_{\mathcal{A}^3}}{\partial\theta} \le \frac{\partial\Pi_{\mathcal{O}^2\mathcal{A}}}{\partial\theta}\right)\Big|_{\theta=\frac{\pi}{2}} \to \left(\frac{\partial\Pi}{\partial\theta}\Big|_{\xi'=3} < \frac{\partial\Pi}{\partial\theta}\Big|_{\xi'=1}\right)\Big|_{\theta=\frac{\pi}{2}}, \tag{S52}$$

which can be simplified to

$$f_x\left(\frac{10}{3} + \sqrt{3}\frac{\cot\phi}{\sin\theta}\right) + f_y\left(\frac{2}{\sqrt{3}} - \frac{\cot\phi}{\sin\theta}\right) \le 10nk\bar{b}\pi\frac{\sqrt{\left(\frac{2\sin\phi\sin\theta}{3}\right)^2 + \cos^2\phi}}{\sin^2\phi\sin\theta}. \tag{S53}$$

Here, the equality sign represents line IV in Supplementary Fig. 12. There is a certain ratio that induces the metamaterial to deform into its $\mathcal{A}^3$ mode (line V in Supplementary Fig. 12). The boundaries defined by Eq. (S49) and (S51) intersect the boundary of the kinematic bifurcation at a point with the load ratio given by

$$\frac{f_x}{f_y} = \frac{\begin{vmatrix} 2 & \frac{2}{\sqrt{3}}+\frac{\cot\phi}{\sin\theta} \\ 10 & \frac{2}{\sqrt{3}}-\frac{\cot\phi}{\sin\theta} \end{vmatrix}}{\begin{vmatrix} \frac{2}{3}-\sqrt{3}\frac{\cot\phi}{\sin\theta} & 2 \\ \frac{10}{3}+\sqrt{3}\frac{\cot\phi}{\sin\theta} & 10 \end{vmatrix}}, \tag{S54}$$

where | | denotes the determinant. Eq. (S54) can be reduced to

$$\frac{f_x}{f_y} = \frac{\frac{4}{\sqrt{3}}+3\frac{\cot\phi}{\sin\theta}}{3\sqrt{3}\frac{\cot\phi}{\sin\theta}}, \tag{S55}$$

and at the bifurcation instant $\theta = 90°$ to

$$\frac{f_x}{f_y} = \frac{\frac{4}{\sqrt{3}}+3\cot\phi}{3\sqrt{3}\cot\phi}. \tag{S56}$$

As per the case of N_4, the metamaterial locks into its $\mathcal{A}^3$ mode provided that the load ratio of Eq. (S55) is maintained during the reconfiguration (for $\phi = 60°$, this ratio is $\frac{f_x}{f_y} = \frac{7}{3\sqrt{3}} \approx 0.74$). The boundary of the lock configuration in $\mathcal{A}^3$ mode (Line VI in Supplementary Fig. 12) can be obtained from the first variation of Eq. (S40) by setting $\theta = \theta^L$. This boundary can be expressed as

$$3f_x + \sqrt{3}f_y = \frac{12nk\bar{b}(\pi-\theta^L)}{\sin\theta^L} . \frac{\sqrt{\cos^2\phi + \sin^2\phi\left(\frac{2}{3}\sin\theta^L - \cos\theta^L\right)^2}}{\sin^2\phi\left(\frac{2}{3}\sin\theta^L - \cos\theta^L\right)}. \tag{S57}$$

S10. Experiments

S10.1. Base material characterization

To characterize the Young's modulus of the base paperboard material we fabricated rectangular samples of dimensions 240 mm × 5 mm (30 mm on each side for the grip) as suggested in literature [7] for a better outcome than that provided by standardized methods, i.e., ISO 1924–2 (see the left-hand-side of Supplementary Fig. 13). The stress-strain response of the base paperboard material of our proof-of-concept prototype was characterized using in-plane uniaxial tensile tests conducted on rectangular samples. Tests were carried out under in-plane uniaxial tension along two main directions, i.e., machine direction (MD) and cross direction (CD) (Supplementary Fig. 13). The results show that the tensile Young's modulus and strength of our paperboard sheets in the MD direction is about 7.9 GPa and 57 MPa, respectively, roughly

two times higher than the values obtained in the CD direction. The difference we observe can be attributed to the pulp and paper process which tends to align the network of cellulose fibers in the MD direction.

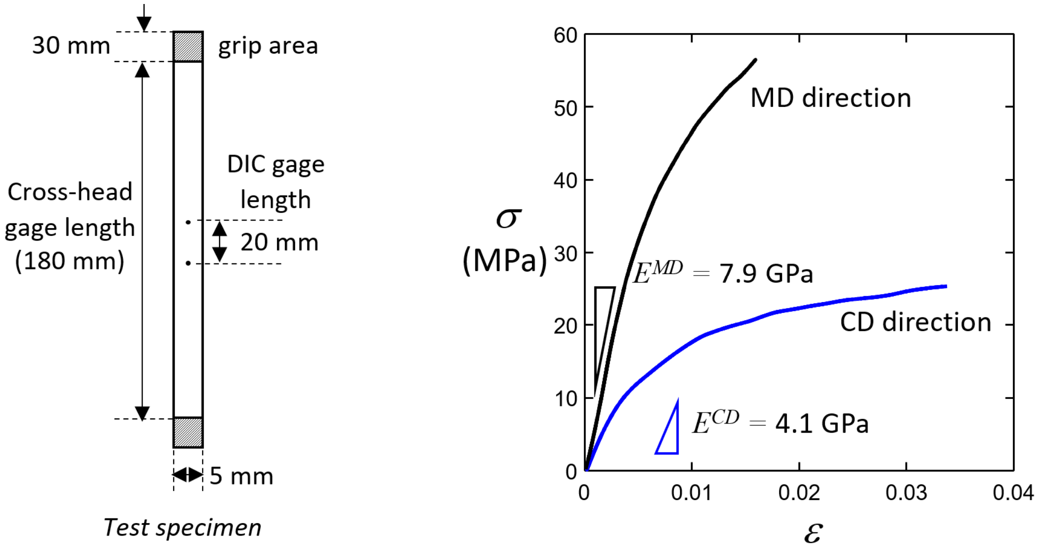

Supplementary Fig. 13 Tensile test specimen dimensions and results of paperboard. Specimen geometry of base paperboard material along with their uniaxial stress-strain responses in the machine direction (MD) and cross (CD) direction.

S10.2. Response of periodic cellular material

Prior to carrying a series of experiments on our material system, we studied the properties of finite size specimens to ensure they are representative of those of their periodic counterparts. We study the role of in-plane and out-of-plane tessellation of our material building block with the goal of determining the smallest material system (minimum number of unit-cells in all three directions) that represents the response of an unbounded periodic material.

To perform our convergence analysis of tessellation level, we examine two representative units, $\widehat{\mathrm{N}}_4\mathrm{n}_n$ and $\widehat{\mathrm{N}}_6\mathrm{n}_n$, and adopt the notation $(l_{\mathbf{e}_1}, l_{\mathbf{e}_2})$ to refer to an in-plane level of tessellation where the primitive unit is tessellated $l_{\mathbf{e}_1}$ times in the in-plane direction $\mathbf{e}_1$ and $l_{\mathbf{e}_2}$ times in $\mathbf{e}_2$. Given the role of tessellation along the three directions, we denote our material system as $\widehat{\mathrm{N}}_N\mathrm{n}_n(l_{\mathbf{e}_1}, l_{\mathbf{e}_2})$, and study two representative systems, $\widehat{\mathrm{N}}_4\mathrm{n}_6(7{,}7)$ and $\widehat{\mathrm{N}}_6\mathrm{n}_6(7{,}7)$. The out-ot-plane tessellation is assessed by the number of layers *n* stacked in the third direction. Our experiments investigate:

(A) In-plane tessellation. For a single layer sample $\widehat{\mathrm{N}}_4\mathrm{n}_1$, determine the minimum number of in-plane unit cells that is representative of the unbounded in-plane tessellation.

(B) Out-of-plane tessellation. For a multilayer specimen $\widehat{\mathrm{N}}_4\mathrm{n}_n$ with minimum number of in-plane unit cells, assess the minimum number of stacking layers, n, that can parallel the response of the unbounded periodic domain.

(C) Comparison between of $\widehat{\mathrm{N}}_4\mathrm{n}_1$ and $\widehat{\mathrm{N}}_6\mathrm{n}_1$ at given levels of tessellation.

(A) Mechanical response of single-layer specimen. Supplementary Fig. 14**a** shows the primitive unit-cell of the locked unit $\widehat{\mathrm{N}}_4\mathrm{n}_1$ along with the effective area of four tessellation levels (1,1), (2,2), (3,3) and (4,4), each shown by a color ranging from brown to yellow. In Supplementary Figs. 14**b**, **c**, the statistical values of Young's moduli and yield strength (as measured in the subset of Supplementary Fig. 14**b**) of a single layer sample show that by increasing the number of unit-cells, the convergence for both parameters occur at the tessellation level of (7,7). Beyond this level, negligible changes in properties can be observed. (7,7) can thus be considered as the minimum level of tessellation representing the periodic unbounded response.

(B) Mechanical response of multilayered specimens. The role of layer stacking (*z*-direction) was assessed for a specimen with (7,7) tessellation level (Supplementary Fig. 14**d**). The results in Supplementary Figs. 14**e**, **f** show that adding *n* layers results in a convergence of the Young's modulus and strength at n = 6. Beyond this value, only marginal changes can be observed, indicating that the periodic response of $\widehat{\mathrm{N}}_4\mathrm{n}_6$ with tessellation level (7,7) is attained with a minimum of 6 layers stacked in the *z*-direction.

(C) Comparison of the mechanical response of $\widehat{\mathrm{N}}_4\mathrm{n}_1$ *and* $\widehat{\mathrm{N}}_6\mathrm{n}_1$ *at given levels of tessellation.* Supplementary Fig. 14**g** shows the primitive unit cell of the locked unit kinematic chain $\widehat{\mathrm{N}}_6\mathrm{n}_1$ along with the manufactured paper sample with a tessellation level (7,7). Supplementary Fig. 14**h**, **i** show respectively the Young's modulus and the yield strength of the single layer $\widehat{\mathrm{N}}_6\mathrm{n}_1$ and compare them with those of $\widehat{\mathrm{N}}_4\mathrm{n}_1$ at given levels of tessellation. The results show that a single unit chain, i.e., tessellation level (1,1), has the highest stiffness, but the lowest strength. Upon increasing the tessellation level up to (7,7), the stiffness decreases for both units; on the other hand, for $\widehat{\mathrm{N}}_4\mathrm{n}_1$ the strength monotonically increases, while for $\widehat{\mathrm{N}}_6\mathrm{n}_1$ we observe first an increase and then a decrease. Beyond the tessellation level (7,7) the mechanical properties tested in our experiment do not show noticeable changes (Supplementary Fig. 14**h**, **i**). Hence (7,7) is the minimum level of tessellation that we identify as representative of the unbounded periodic response.

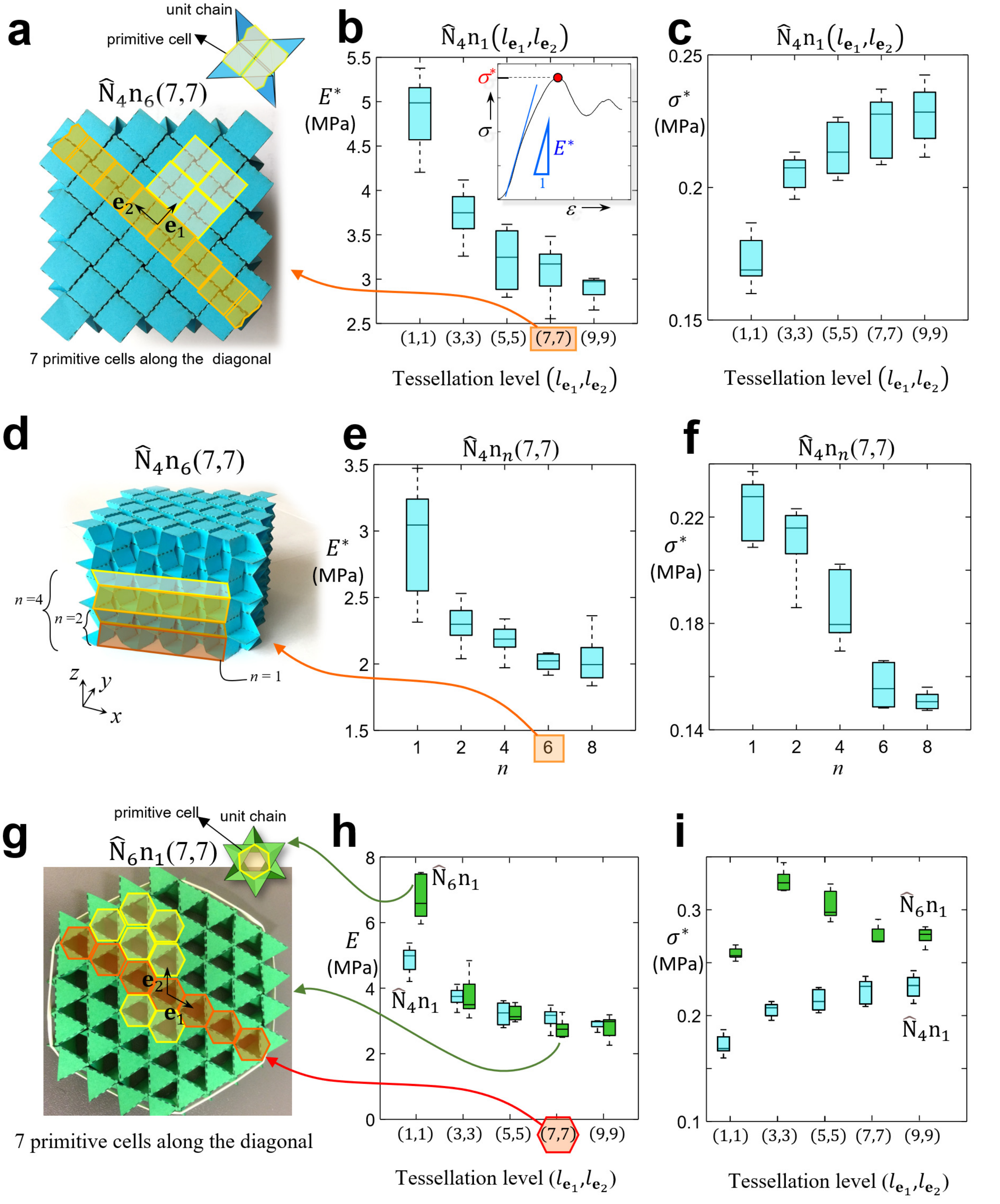

Supplementary Fig. 14 Measured properties of finite size specimens. Fabricated samples and measured properties of $\widehat{N}_4n_n$ unit locked in mode $\mathcal{A}^2$; statistical data for each given geometry are test results of five samples. **a** Top view illustration of manufactured unit chain showing its unit chain and tessellation levels. Compressive Young's modulus **b** and yield strength **c** for a sample with one layer only and given planar tessellation levels. The subset in **b** schematically illustrates how the compressive Young's modulus E^* and yield strength σ^* were measured. **d** Material system $\widehat{N}_4n_6$ with representative number, n, of stacked layers. Measured compressive Young's modulus E^* **e** and yield

strength σ^* **f** for given numbers of stacking layers n. **g** Top view of representative fabricated $\widehat{\mathrm{N}}_6\mathrm{n}_1$ sample with unit chain area in locked mode $\mathcal{A}^3$ and tessellation level (7,7). Yellow hexagons represent the top area of our primitive unit cells used for the calculation of the stress values. Compressive Young's modulus **h** and yield strength **i** of $\widehat{\mathrm{N}}_6\mathrm{n}_1$ and $\widehat{\mathrm{N}}_4\mathrm{n}_1$ at given tessellation levels. For each tessellation level, five tests were conducted.

S11. Manufacturability

Most of existing reconfigurable metamaterial concepts using origami concepts require a laborious process of fabrication. The reason lies mainly in the non-developable nature of their patterns [8-10], which often adds an additional layer of manufacturing sophistication. In several cases, the fabrication of a single three-dimensional unit cell requires several steps (cutting, folding, and gluing) [8, 9]. The spatial assembly of multiple unit cells is another elaborate undertaking, as each cell needs to be attached one by one in space to its neighbor via edge-to-edge bonding [8-11]. While 3D printing has been effectively used to fabricate origami-based materials [10,12-14], the high stiffness of the 3D printed hinges has been shown to sacrifice their foldability [10,12,13]. Additionally, 3D printing has been used mainly with soft materials [13, 14], thereby bringing to realization prototypes with limited load-bearing capacity.

In contrast, our crease and cut patterns are fully developable. This enables us to use a relatively straightforward process entailing two basic steps of fabrication which can be easily automated. From a single layer of the base material, we introduce in one step: the tessellation of shaped voids and folding lines through laser cutting. After partially folding one kirigami layer along the origami crease pattern, we stack identical layers and bond (here, using glue) their triangular faces. This process does not require the assembly of 3D unit cells in space, rather three-dimensionality is imparted in the flat configuration where the constituent layers are stacked and bonded together. As a result, the flat multilayered crease pattern can be easily folded in space since each layer is geometrically constrained to lay between parallel planes (Supplementary Movie 1). Such characteristics enable to bypass the more laborious procedures currently pursued in the literature.

Supplementary References

[1] Pellegrino, S., & Calladine, C. R. Matrix analysis of statically and kinematically indeterminate frameworks. *Int. J. Solids Struct.* **22**, 409-428 (1986).

[2] Pellegrino, S. Structural computations with the singular value decomposition of the equilibrium matrix. *Int. J. Solids Struct.* **30**, 3025-3035 (1993).

[3] Kumar, P., & Pellegrino, S. Computation of kinematic paths and bifurcation points. *Int. J. Solids Struct.* **37**, 7003-7027 (2000).

[4] Klima, R., Sigmon, N., & Stitzinger, E. *Applied Abstract Algebra with MapleTM and MATLAB®: A Maple and MATLAB Approach* (Vol. 34) (CRC Press, 2015).

[5] Mordell, L. J. *Diophantine equations*. (Academic Press, 1969).

[6] Wagner, M. A., Lumpe, T. S., Chen, T., & Shea, K. Programmable, active lattice structures: Unifying stretch-dominated and bending-dominated topologies. *Extreme Mech. Lett.* **29**, 100461 (2019).

[7] Yoshihara, H., & Yoshinobu, M. Effects of specimen configuration and measurement method of strain on the characterization of tensile properties of paper. *J. Wood Sci.* **60**, 287-293 (2014).

[8] Overvelde, J. T., De Jong, T. A., Shevchenko, Y., Becerra, S. A., Whitesides, G. M., Weaver, J. C., Hoberman, C., & Bertoldi, K. A three-dimensional actuated origami-inspired transformable metamaterial with multiple degrees of freedom. *Nat. Commun.* **7**, 1-8 (2016).

[9] Overvelde, J. T., Weaver, J. C., Hoberman, C., & Bertoldi, K. Rational design of reconfigurable prismatic architected materials. *Nature* **541**, 347-352 (2017).

[10] Filipov, E. T., Tachi, T., & Paulino, G. H. Origami tubes assembled into stiff, yet reconfigurable structures and metamaterials. *Proc. Natl. Acad. Sci. USA* **112**, 12321-12326 (2015).

[11] Schenk, M., & Guest, S. D. Geometry of Miura-folded metamaterials. *Proc. Natl. Acad. Sci. USA* **110**, 3276-3281 (2013).

[12] Cheung, K. C., Tachi, T., Calisch, S., & Miura, K. Origami interleaved tube cellular materials. *Smart Mater. Struct.* **23**, 094012 (2014).

[13] Zhao, Z., Kuang, X., Wu, J., Zhang, Q., Paulino, G. H., Qi, H. J., & Fang, D.. 3D printing of complex origami assemblages for reconfigurable structures. *Soft Matter* **14**, 8051-8059 (2018).

[14] Lin, Z., Novelino, L. S., Wei, H., Alderete, N. A., Paulino, G. H., Espinosa, H. D., & Krishnaswamy, S. Folding at the Microscale: Enabling Multifunctional 3D Origami-Architected Metamaterials. *Small* **16**, 2002229 (2020).
