# Supplementary material for: Rigidly flat-foldable class of lockable origami-inspired metamaterials with topological stiff states
Source: Nat Commun. 2022 Apr 5;13:1816. doi: 10.1038/s41467-022-29484-1 (PMC8983707; doi:10.1038/s41467-022-29484-1)
Supplement: Supplementary file 3 — Description of Additional Supplementary Files [file 41467_2022_29484_MOESM3_ESM.pdf]

Description of Additional Supplementary Files

File Name: Supplementary Movie 1
Description: Manufacturing

File Name: Supplementary Movie 2
Description: Folding modes of N_4n_6 prototype

File Name: Supplementary Movie 3
Description: Folding modes of N_6n_6 prototype

File Name: Supplementary Movie 4
Description: Kinematic paths of N_4n_4

File Name: Supplementary Movie 5
Description: Kinematic paths of N_6n_4

File Name: Supplementary Movie 6
Description: Kinematic paths of N_8n_5

File Name: Supplementary Movie 7
Description: Kinematic paths of $N_{10}n_5$

File Name: Supplementary Movie 8
Description: Biaxial compression of N_4n_4 when $f_x > f_y$

File Name: Supplementary Movie 9
Description: Biaxial compression of N_4n_4 when $f_y > f_x$

File Name: Supplementary Movie 10
Description: Biaxial compression of N_4n_4 when $f_x = f_y$

File Name: Supplementary Movie 11
Description: Load-bearing capacity of N_4n_6 prototype

File Name: Supplementary Movie 12
Description: Load-bearing capacity of N_6n_6 prototype
